# Functional Compound Bioaccessibility and Microbial Viability in Green and Black Tea Kombucha During Simulated Digestion

**DOI:** 10.3390/foods14162770

**Published:** 2025-08-09

**Authors:** Gloria Ghion, Jacopo Sica, Sofia Massaro, Armin Tarrah, Tove Gulbrandsen Devold, Davide Porcellato, Alessio Giacomini, Frederico Augusto Ribeiro de Barros, Viviana Corich, Chiara Nadai

**Affiliations:** 1 Department of Agronomy Food Natural Resources Animal and Environment (DAFNAE), University of Padova, 35020 Padova, Italy; gloria.ghion@phd.unipd.it (G.G.); jacopo.sica@unipd.it (J.S.); sofia.massaro@phd.unipd.it (S.M.); viviana.corich@unipd.it (V.C.); chiara.nadai@unipd.it (C.N.); 2 Canadian Research Institute for Food Safety, Department of Food Science, University of Guelph, Guelph, ON N1G 2W1, Canada; atarrah@uoguelph.ca; 3 Faculty of Chemistry, Biotechnology and Food Science, Norwegian University of Life Sciences, 1432 Ås, Norway; tove-gulbrandsen.devold@nmbu.no (T.G.D.); davide.porcellato@nmbu.no (D.P.); 4 Interdepartmental Centre for Research in Viticulture and Enology (CIRVE), University of Padova, 31015 Conegliano, Italy; 5 Department of Food and Technology, Universidade Federal de Viçosa, Viçosa 36570-900, Brazil; fredbarros@ufv.br; 6 Department of Land, Environment, Agriculture and Forestry (TESAF), University of Padova, Viale dell’Università 16, 35020 Legnaro, Italy

**Keywords:** kombucha, fermentation, antimicrobial activity, antioxidant activity, free amino acids, L-theanine

## Abstract

Kombucha, a fermented tea beverage, is gaining popularity due to its rich content of bioactive compounds and associated health benefits. Kombucha fermentation involves a complex microbial consortium, including acetic acid bacteria, lactic acid bacteria, and yeasts, that works synergistically to enhance its nutritional and functional properties. Key compounds produced during fermentation provide antioxidant, anti-inflammatory, and antimicrobial benefits. Despite its well-documented health-promoting properties, limited research exists on how human digestion influences the stability and functionality of kombucha bioactive components. This study investigated how digestion impacts kombucha made from green and black teas, focusing on free amino acid content, antioxidant activity, antimicrobial potential, and microbiota viability. Results showed that digestion significantly increased free amino acids, as fermentation released peptides suitable for gastrointestinal digestion. However, L-theanine, a beneficial tea compound, was no longer detectable after fermentation and digestion, suggesting limited bioaccessibility. Digested kombucha exhibited higher antioxidant activity and stronger antimicrobial effects compared to undigested tea. Moreover, culture-dependent and PMA-based sequencing confirmed the survival of viable microbial strains through simulated gastrointestinal conditions, suggesting the potential of kombucha as a source of live, functional microbes. These findings support the role of kombucha as a natural functional beverage whose health benefits not only persist but may be enhanced after digestion.

## 1. Introduction

Kombucha is a tea-based fermented beverage that has received considerable attention in recent years from consumers and researchers as a functional beverage due to its high content of bioactive compounds. The potential health benefits of kombucha are largely attributed to its primary ingredient, tea, which is naturally rich in polyphenols and possesses strong antioxidant properties. Numerous studies demonstrated that tea polyphenols exert antioxidant, antibacterial, and anti-inflammatory effects when consumed as part of the human diet. These beneficial effects are mainly linked to the polyphenolic compounds of tea, particularly catechin, that make up about 70–80% of the total polyphenol content in green tea and 20–30% in black tea [1,2]. The major catechins include catechin (C), epicatechin (EC), epicatechin 3-O-gallate (ECG), epigallocatechin (EGC), epigallocatechin 3-O-gallate (EGCG), and gallic acid (GA).

The fermentation process is driven by a complex microbial consortium, including acetic acid bacteria (AAB; mainly *Komagataeibacter*, *Gluconobacter*, and *Acetobacter* species), lactic acid bacteria (LAB; mainly *Lactobacillus* and *Lactococcus*), and various yeasts (*Zygosaccharomyces*, *Brettanomyces*, *Saccharomyces*, *Schizosaccharomyces*, *Saccharomycodes*, and *Torulaspora*, among others), that establishes a strong symbiotic relationship [3,4,5,6,7]. Kombucha origins track back to around 220 B.C. in Asia, and it is now largely consumed for its nutraceutical properties [8,9]. During the fermentation process, which lasts 5–10 days at 20–30 °C, the tea composition, including polyphenols, flavonoids, vitamins, and essential minerals, is enriched in several organic acids, including acetic, glucuronic, and gluconic acids, that lower the pH, preventing bacterial contamination. The microbiological process starts with the hydrolysis of sucrose into glucose and fructose by osmophilic yeasts, which are then utilized by AAB to produce cellulose and organic acids, while also enriching the kombucha with various bioactive compounds [10,11,12]. Several studies, in vitro or in animal models, focused on the beneficial activities of this fermented tea that contribute to the increased nutritional, antioxidant, anti-inflammatory, and anti-obesity hypocholesterolemic effects, together with antimicrobial activity against foodborne and human pathogens [9,13,14,15,16].

In the literature, kombucha is frequently referred to as a probiotic drink due to the presence of live and potentially beneficial microorganisms [17,18,19,20]. Many commercial products are labeled with claims such as “natural”, “living culture”, “non-dairy probiotics”, or “healthy for your gut” [21]. However, these claims are often unsubstantiated and not supported by rigorous scientific evidence [22,23,24]. While kombucha does contain live microorganisms, the presence of viable microbes alone is not sufficient to define it as a probiotic product. According to international definitions, probiotic strains must be well-characterized, show demonstrated health benefits, and survive passage through the gastrointestinal tract [9,25]. However, microbial viability and the potential probiotic role of kombucha-associated strains remain largely unexplored.

Moreover, only a few studies investigated the impact of digestion on the stability and functionality of kombucha’s bioactive compounds [26,27,28]. No information is currently available on the release of amino acids or on the antimicrobial activity of kombucha following digestion. Therefore, this study investigated the effect of digestion on the bioaccessibility of amino acids and antioxidant and antimicrobial properties and the survival of viable microorganisms, providing novel insights into the functional potential of kombucha after gastrointestinal passage [29,30,31,32,33,34,35].

## 2. Materials and Methods

### 2.1. Kombucha Production

Green tea (Lung Ching variety), black tea (Darjeeling Gielle FTGFOP1 Second Flush variety), and the cellulosic biofilm known as symbiotic culture of bacteria and yeast (SCOBY) were purchased from the Departamento de Tecnologia de Alimentos, Universidade Federal de Viçosa, Viçosa, Brazil. Kombucha was produced from green and black tea leaves according to the methodology used by Cardoso et al. [36]. Initially, 50 g of sucrose were dissolved in 1 L of sterile water. Green tea (GT) and black tea (BT) were infused at a concentration of 12 g/L in the water at 95 °C for 4 min and 75 °C for 1 min, respectively. After infusion, the mixture was strained through a cotton gauze, poured into sterile glass jars, and let to cool to approximately 25 °C. Then, 3% (*w*/*v*) of SCOBY and 100 mL/L of a previously made kombucha batch were added to reduce pH and inhibit the growth of undesirable microorganisms. The opening of the jar was covered with a clean cotton cloth and fermentation was carried out in the dark at 25 ± 2 °C for 5 days for green kombucha (GK) and 7 days for black kombucha (BK). Upon completion of fermentation, the SCOBY was removed and kombucha samples were centrifuged at 15,000× *g* for 15 min and stored at –20 °C until further use. BT and GT were prepared using the same tea and sugar concentrations but without the addition of the SCOBY and kombucha.

### 2.2. The pH Determination and Chemical Composition

The pH was determined by a calibrated bench-top pH meter (Hanna Instruments, Inc. Woonsocket, (RI), USA). The pH was measured for both GK and BK at the beginning of the fermentation and at the end of the fermentation after 5 days and 7 days, respectively. The pH of the unfermented teas (BT and GT) was measured after the infusion step. Moisture content (method AOAC 934.01), dry matter, and ash (method AOAC 942.05) were determined following standard procedures [37]. The crude protein content was quantified with the Kjeldahl method (method AOAC 2001.11) [38]. Ash and crude protein values were expressed as g/100 mL as a dry weight basis. Sugars were measured using Enzytec™ Liquid Sucrose/D-Glucose/D-Fructose E8190 (R-Biopharm, Milan, Italy). The final concentrations of sugars were reported as g/L, and all measurements were performed in triplicate.

### 2.3. In Vitro Digestion with the INFOGEST Static Model

Liquid samples of kombucha and tea were freeze-dried under vacuum for 5 days using a freeze dryer (Modulyo, Edwards, West Sussex, UK). Lyophilized samples were then resuspended in a defined amount of sterile water to achieve a final concentration of 0.3% crude protein to standardize the digestion.

A static digestion protocol was conducted on 0.5 mL of the resuspended kombucha and tea samples based on the harmonized INFOGEST 2.0 model [39]. Samples were exposed to pepsin and pancreatin enzymes, with salivary amylase and gastric lipase enzymes omitted due to the absence of starch and lipids in the samples [40]. In addition, pancreatin was subjected to 5 min sonication, followed by 3000× *g* centrifugation for 5 min at RT prior to the addition of the supernatant in the intestinal phase [41]. The inclusion of the centrifugation step was reported not to significantly affect the pancreatic activity, as reported by Tanambell et al. [41]. Enzyme activities and bile concentration were measured prior to the digestion experiment, as described in the harmonized protocol, with the abovementioned sonication and centrifugation included in the pancreatin activity measurement. Simulated salivary fluid (SSF), simulated gastric fluid (SGF), and simulated intestinal fluid (SIF) were prepared, as described by the protocol. A preliminary digestion simulation was performed to determine the amount of HCl and NaOH required to adjust the pH for the gastric (pH 2) and intestinal (pH 7) phases, respectively. All simulated fluids (SSF, SGF, SIF) were pre-incubated at 37 °C to simulate the body temperature before starting the procedure. The different digestion phases were conducted in an Ecotron incubator shaker (Infors HT, Switzerland) at 160 rpm agitation to mimic the intestinal peristaltic movements similar to those achieved in each digestive compartment [42]. In the oral phase, kombucha and tea samples were diluted 1:1 (*v*/*v*) with SSF, 0.3 M CaCl_2_, and Milli-Q water (including volume replacement for amylase). After 2 min of incubation at 37 °C with agitation, the oral bolus was diluted at a volumetric ratio of 1:1 with SGF, 0.3 M CaCl_2_, gastric pepsin (2000 U/mL final volume), 1 M HCl, and Milli-Q water. Afterwards, the gastric digestion was carried out for 2 h of incubation at 37 °C with agitation. Subsequently, for the intestinal digestion, the kombucha and tea samples from the gastric step were diluted at a ratio of 1:1 (*v*/*v*) with SIF, 0.3 M CaCl_2_, bovine bile (10 mM), and pancreatin (100 U of trypsin activity/mL final volume). The pH was adjusted to 7 and the intestinal phase was carried out for 2 h under the conditions described above. The proteolytic digestive enzymes were then irreversibly inhibited by adding Pefabloc^®^ SC (4-(2-aminoethyl) (Bottmingen, Switzerland) benzenesulfonyl fluoride) at a final concentration of 5 mM. After the addition of Pefabloc^®^, samples were immediately aliquoted and frozen at −20 °C until further analysis. The digestions were performed in triplicate. A control sample (hereafter referred to as “enzyme blank”), which consisted of the gastrointestinal juices, enzymes, and water in place of tea or kombucha, was included in the experimental trials to observe the potential presence of enzymatic auto-hydrolysis and used as background [43].

### 2.4. Degree of Hydrolysis (OPA) After In Vitro Digestion

The O-phthalaldehyde (OPA) spectrophotometric assay was used to measure the extent of peptide bond hydrolysis in tea and kombucha, both before and after a complete in vitro gastrointestinal digestion [44]. The OPA buffer was prepared according to Goodno et al. [45] as follows: 25 mL of 100 mM di-Na-tetraborate and 5 mL of 10% (*w*/*w*) Na-dodecyl-sulfate (SDS) were mixed with 40 mg of OPA, previously dissolved in 1 mL of methanol, to achieve a final OPA concentration of 6 mM. Then, 100 μL of β-mercaptoethanol (BME) were added to the solution, the volume was brought to 50 mL with water, and pH was adjusted to 9. The OPA reagent was freshly made for every experiment. Samples were centrifuged at 4000× *g* for 10 min (Micromax, IEC model, International Equipment Company (IEC), Needham, MA, USA), and the supernatants were diluted (1:100) with buffer (OPA buffer without OPA and BME). Then, 50 µL of the diluted samples and ultrapure water (blank) were pipetted onto a 96-well plate and mixed with 200 µL of OPA buffer. The plate was incubated at room temperature for 2 min, shaken for 5 secs, and read at 340 nm using a microplate reader. The concentration of free N-terminal was determined using a standard calibration curve of 0.5–3 mM L-leucine and expressed as mM L-leucine equivalent (LE). The assay was performed using three biological and three technical replicates.

### 2.5. HPLC Analysis of Free Amino Acid

The proportion of free amino acids (FAA) released from the protein in the products and solubilized into the digestive fluids (bioaccessibility) was determined by high-performance liquid chromatography (HPLC) with post-column with derivatization using OPA. FAA were quantified by adding 0.5 mL of internal standard solution (0.1 M HCl; 0.4 µmol/mL L-norvalin; Sigma, St. Louis, MO, USA) to 0.5 mL of sample. Samples were sonicated for 30 min and then centrifuged (Thermo Scientific, Heraeus Multifuge X3R, Thermo Fisher Scientific, Dreieich, Germany) at 2500× *g* for 40 min at 4 °C. A total of 1 mL of 4% trichloroacetic acid (TCA, Sigma) was added to 1 mL of supernatant, mixed in a mini shaker (Gene 2, New York, NY, USA), and placed in ice for 30 min. After centrifugation at 15,600× *g* for 5 min at 4 °C, the samples were filtered (0.22 µm cellulose acetate filter, Advantec, Dublin, CA, USA) and stored at –20 °C until analysis. Before separation, 350 µL of borate buffer (0.4 M, pH 10.2, Agilent Technologies) was added to 50 µL of sample. Separation of AA was performed using an Agilent series 1200 pump (Agilent Technologies, Singapore) and an Agilent 1200 series HPLC system (Agilent Technologies, Singapore) consisting of a pump, autosampler, column oven, thermostat, and fluorescence detector. The system was driven by OpenLAB CDS (Agilent Technologies) software (version 2.4). An XTerra RP 18 column (150 × 4.6 mm; Waters, Milford, MA, USA) was used for separation of AA at 42 °C. Derivatization with OPA was performed according to Bütikofer and Ardö [46]. The enzyme blank was included in the analysis. The analysis of the FAA was performed on three biological replicates and with three technical replicates.

### 2.6. Determination of Antioxidant Capacity

The antioxidant activity of kombucha and tea samples after in vitro digestion was measured using the spectrophotometric method using synthetic radical DPPH (2,2-diphenyl-1-picryl-hydrazyl). Samples were filtered through a 0.22 μm PTFE filter and 5 μL of digested sample or solvent blank (96% ethanol) were mixed with 2 mL DPPH reagent prepared at a concentration of 25 µg/mL in 96% ethanol. All chemicals were purchased from Merck (Darmstadt, Germany). Samples were incubated in the dark for 30 min, and the absorbance was then measured at 515 nm. The antioxidant activity of kombucha and tea samples was expressed by the percentage of DPPH inhibition using the following formula:

$$\%\;\mathrm{inhibition}\;=\;\frac{A0\;-\;\mathrm{As}\;}{A0}\;\times \;100$$

where A0 represents the absorbance of DPPH solution at 515 nm without tested sample (blank), and As represents the absorbance of DPPH solution at 515 nm with tested sample. All assays were performed in triplicate.

### 2.7. Antimicrobial Activity

The pathogenic bacteria that were used for the antimicrobial activity test included *Salmonella enterica* subsp. *enterica* serovar Typhimurium SL1344 (firstly isolated from cattle), *Yersinia enterocolitica* Xen24 (an engineered strain for pathogenicity research purposes), *Shigella sonnei* ATCC 11060 (isolated from a human source), *Listeria monocytogenes* EGD-e (ATCC BAA-679) (isolated from rabbit), and the enterotoxigenic CFA-i (ETEC) *Escherichia coli* strain, which was isolated from infants with diarrhea. Moreover, the antimicrobial activity of the digested samples was assessed against *Lactiplantibacillus plantarum*, a LAB commonly found in the human digestive tract, to evaluate potential inhibitory effects on the growth of gut microbes. The bacterial strains were obtained from the departmental culture collection of the Norwegian University of Life Sciences (NMBU) in Ås (Norway). Each bacterial strain was cultured in BHI broth (Oxoid Limited, Basingstoke, Hampshire, UK) for pathogenic bacteria and MRS for LAB and then incubated at 37 °C for 24 h. To estimate CFU/mL in each sample, 0.1 mL of each strain was plated on BHI agar (for pathogenic bacteria) or MRS agar (for LAB) and incubated at 37 °C for 24 h and 48 h, respectively. Based on the obtained counts, the strains were subsequently diluted in sterile broth to achieve a final concentration of 10^5^ CFU/mL.

The antimicrobial activity of digested kombucha and tea samples was evaluated and compared as follows. The digested samples were centrifuged at 15,000× *g* for 15 min and sterilely filtered (0.22 μm, Millipore, Merck, Burlington, MA, USA). For the antimicrobial assay, 240 µL of each bacterial suspension at a concentration of 10^5^ CFU/mL in fresh BHI/MRS was added to wells of a sterile 96-well microtiter plate containing 60 μL of digested sample (tea or kombucha). The chosen digested extract volume was 20% of the total volume: this percentage represents a good compromise among the extract dose, the turbidity caused by the extract, and the degree of dilution of the growth medium. Samples were standardized before digestion, following the standard protocols of INFOGEST, in order to make the tea and kombucha samples comparable and to compare the properties of the digested samples as they are.

For each run, both growth and sterility controls were included. The growth control contained 240 μL of fresh BHI/MRS and 60 μL of bacterial suspension, while the sterility control contained 240 μL of fresh BHI/MRS and 60 μL of sterile water. For each combination of bacterial strain and digested sample, a blank was included to account for any interference from the digested sample. The blank consisted of 240 μL of fresh BHI/MRS and 60 μL of sterile sample. All wells had a final volume of 300 μL. Antimicrobial testing was performed in a 96-well microtiter plate, and all samples were run in triplicates. Plates were incubated at 37 °C for 24 h, and the optical density at the absorbance of 600 nm (OD_600_) was recorded every 15 min (Supplementary Table S1) using a Multiskan SkyHigh Microplate Spectrophotometer (Thermo Fisher Scientific, Waltham, MA, USA). Results were calculated by subtracting the blank OD from the sample OD.

### 2.8. Viability Assessment by Culture-Based Methods

Viability tests were conducted at different stages of the in vitro digestion: before digestion (T0), after exposure to simulated gastric fluid (SGF), and after 60 and 120 min of simulated intestinal fluid (SIF) (I 60 and I 120). For each time point, individual sample tubes were processed. One milliliter of sample was aseptically collected and serially diluted in 9 mL phosphate-buffered saline (PBS, pH 7.4). Tubes were kept on ice during dilution. Aliquots were plated on plate count agar (PCA) (Difco™, Franklin Lakes, NJ, USA) for total plate counts. Plates were incubated at 30 °C for 24–48 h.

### 2.9. Viability Assessment by PMA-Modified DNA Sequencing

To evaluate the impact of digestion on microbial viability, samples were treated with the DNA-intercalating dye PMAxx (Biotium, Hayward, CA, USA) prior to DNA extraction. One pellet, obtained by centrifugation and washed in PBS, was resuspended in 500 µL PBS and treated with 1.25 µL PMAxx (10 mM stock) to reach a final concentration of 25 µM. Samples were incubated for 20 min in the dark and then photoactivated for 20 min using a PMA-Lite™ LED Photolysis Device. Biomass was collected by centrifugation (10,000× *g*, 5 min) and subjected to DNA extraction. DNA was extracted using a DNeasy PowerFood Microbial Kit (Qiagen, Düsseldorf, Germany) with a modified protocol that included 15 min of vortexing in step 6 to enhance lysis of difficult-to-disrupt cells [47]. DNA was eluted in 50 µL of elution buffer and quantified with a Qubit 2.0 Fluorometer (Thermo Fisher Scientific, USA). Samples were diluted 10 fold to reduce PCR inhibition. For bacterial profiling, the V3–V4 region of the 16S rRNA gene was amplified using primers Uni340F and Bac806R. For fungal profiling, the ITS1 region of the rRNA operon was amplified using primers ITS1F and ITS2. All PCR reagents and conditions followed Porcellato et al. [48]. Negative controls were included to monitor for contamination during DNA extraction and amplification. Libraries were normalized using the SequalPrep Normalization Plate Kit (Thermo Fisher Scientific), pooled, quantified, and sequenced on an Illumina MiSeq platform at the Norwegian Sequencing Centre (Oslo, Norway).

For community profiling, demultiplexed 300 bp paired-end reads were analyzed with QIIME2 (version 2023.5) [49]. The DADA2 algorithm was used for filtering, trimming, denoising, dereplicating, merging paired reads, and removing chimeric sequences [50]. For bacterial data, the resulting amplicon sequence variants (ASVs) were classified through a Naïve Bayes pre-trained Silva v.138 99% OTUs’ classifier. After the removal of contaminants using blank samples, ASVs were frequency-filtered with a threshold of 5% in at least three samples. The retained sequences were then manually checked using BLAST (version 2.16.0) on NCBI to refine the taxonomic assignment. Fungal ASVs were open-reference clustered against the UNITE database (version 9.0) at a 97% threshold and subsequently classified using the “feature-classifier classify-consensus-vsearch” plugin.

### 2.10. Data Analysis

All experiments were performed in three independent replicas. Data acquired from the treatments and the control groups are presented as mean ± SD. Data visualization and ANOVA with Tukey’s HSD post hoc test were performed using Python libraries’ matplotlib 3.8.0 [51], statsmodels 0.14.1 [52], and seaborn 0.13.2 [53].

In the antimicrobial activity test, the growth curve was obtained by averaging the data of cell turbidity (OD_600_) values of three independent replicates and calculating the difference with respect to the initial value (ΔOD_600_) to allow curves’ comparison. For each growth curve replica, the area under the curve (AUC) was estimated with trapezoidal rule [54].

The modified Gompertz formula [55] was used for microbial growth curve modeling and estimation of the lag phase duration, through the Python library SciPy 1.14.0 [56]. Where the growth pattern was diauxic, only the first phase of the growth was considered.

## 3. Results and Discussion

### 3.1. General Composition of Green and Black Teas and Kombuchas

Chemical and physical properties of BT, BK, GT, and GK were assessed and compared to verify that the kombucha production methodology aligned with parameters typically reported for artisanal kombucha (Table 1).

Statistically significant decreases in pH values were observed in both teas following fermentation, due to increased acidity from organic acids produced during fermentation [57]. This resulted from yeasts metabolizing sucrose into glucose and fructose, transformed by bacteria into various organic acids, such as acetic, lactic, gluconic, as well as glucuronic acid [58,59,60,61]. The pH reduction can improve the bioaccessibility and bioavailability of antimicrobial and antioxidant phenolic compounds, thereby boosting their antimicrobial properties and contributing to enhanced human health [12,62].

After fermentation, dry matter significantly decreased in both kombucha samples (*p* < 0.05). However, ash and protein concentrations remained stable across all samples, indicating that the main fermentation effect was the transformation of the sugars into volatile molecules. The protein values aligned with those previously reported by Kallel et al. [63], who used the Bradford assay to determine the protein concentrations of green and black teas (0.32 g/L in GT and 0.47 g/L in BT). Sucrose concentration in BT and GT was 55.80 ± 0.30 and 57.09 ± 1.24 g/L, respectively. This corresponded to the sucrose added (50 g/L) and a small portion of sucrose already present in the fermentation starter (around 10% *v*/*v*). As expected, fructose and D-glucose were not detected in tea samples, as there was no microbial activity to convert the sucrose. Sugar levels after fermentation are mainly dependent on parameters such as fermentation time, initial sugar concentration, and inoculum size (SCOBY and a portion of already prepared kombucha). After fermentation, BK exhibited markedly higher sucrose content and lower concentrations of fructose and D-glucose compared to GK, despite the extended fermentation duration for BK. This finding is consistent with the results of Cardoso et al. [14], who used the same initial sucrose concentration of 50 g/L, with 10 days of fermentation. This difference is likely attributable to the varying yeast compositions present in the two kombuchas and their invertase activities, which directly influence the fructose and glucose availability [64]. This result suggests that BK and GK developed two distinct microbial communities, influencing the sugar content in each. Overall, in both types of kombucha, D-glucose was consumed more than fructose, indicating that glucose was preferred over fructose as a carbon source by kombucha communities, unless substantial microbial isomerization of glucose into fructose took place in the fermentation medium [63].

### 3.2. Free Amino Acids’ Quantification After In Vitro Digestion

Lyophilized kombucha and tea samples, which had been filtered and resuspended to reach a standardized protein concentration, were analyzed to measure the degree of peptide bond hydrolysis both before and after complete in vitro gastrointestinal digestion, using OPA spectrophotometric assay. This measurement is a practical tool to quantify the free amino groups released as a result of the digestion process.

**Figure 1:**
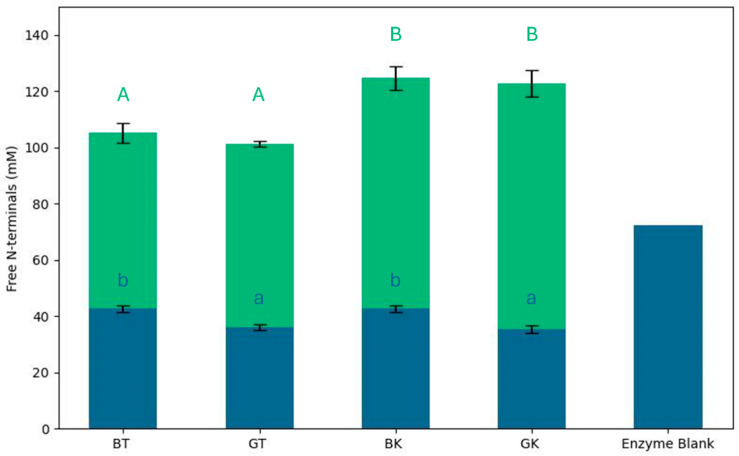
Free *N*-terminal values of gastrointestinal digests of black and green teas and kombucha. Blue bars represent free *N*-terminal concentration of non-digested samples; green bars represent free *N*-terminal concentration of digested samples. Results are expressed as mean of three repetitions, with error bars indicating ± standard deviation. Different lowercase letters indicate statistically significant differences between non-digested samples; different uppercase letters indicate statistically significant differences between digested samples (*p* < 0.05).

The samples included BT, GT, BK, GK, and their respective digested forms: DBT, DGT, DBK, and DGK. The enzyme blank (comprising gastrointestinal juices, enzymes, and water) was also measured (Figure 1).

Self-digestion was observed to a high extent in the enzyme blank, as already observed by Tanambell et al. [41], and it might contribute to the amount of free *N*-terminals. Autolysis of pancreatin under INFOGEST-recommended conditions was previously studied by Atallah et al. [65], and this happened particularly if a depletion of cleavable sites in the food material occurs. In this work, a standard protocol was used with a protein–enzyme ratio that minimized this effect. Moreover, an equal amount of proteins was digested for each sample, allowing samples’ comparison.

The degree of hydrolysis, expressed in terms of free amino groups (mM L-leucine equivalent), significantly increased in all samples after digestion (113.45 ± 11.31 mM L-leucine equivalent).

The BT free *N*-terminals value (42.64 ± 1.21 mM L-leucine equivalent) was significantly higher than that of GT (36.06 ± 1.13 mM L-leucine equivalent), indicating that the availability of amino acids, peptides, and proteins differed due to the tea composition. After fermentation, this concentration was not statistically different in BK and GK (42.64 ± 1.21 and 35.49 ± 1.30 mM L-leucine equivalent, respectively) compared to the respective original matrix.

After digestion, an increase in free *N*-terminals’ values was observed in all the samples, and it was significantly higher in kombucha samples than in tea samples. The reduced hydrolysis of peptide bonds after the digestion of tea samples was probably linked to the presence of phenolic compounds. He et al. [66] demonstrated that tea polyphenols participate in complexing reactions with proteins. Tea polyphenols affect digestive enzyme activity by binding to and precipitating these enzymes, which, in turn, reduce the efficiency of food digestion [67]. The inhibitory effects on digestive enzymes could be due to the combined influence of hydrophobic interactions and hydrogen bond formation between polyphenols and the enzymes [66,68]. This interaction limits the enzymatic hydrolysis of proteins, which, in turn, reduces the release of detectable amino acids after digestion. It was demonstrated that the microorganisms in kombucha can modify the phenolic components of tea, such as catechins. This could explain the greater digestion of the protein and peptide components in kombucha compared to the original tea [59].

Moreover, during kombucha fermentation, an increase in protein concentration was reported, around 6–10 days of fermentation [59], suggesting a different protein composition of the fermented samples. This finding suggests that the fermentation process in kombucha may enhance the susceptibility of peptide bonds to hydrolysis during digestion.

**Figure 2:**
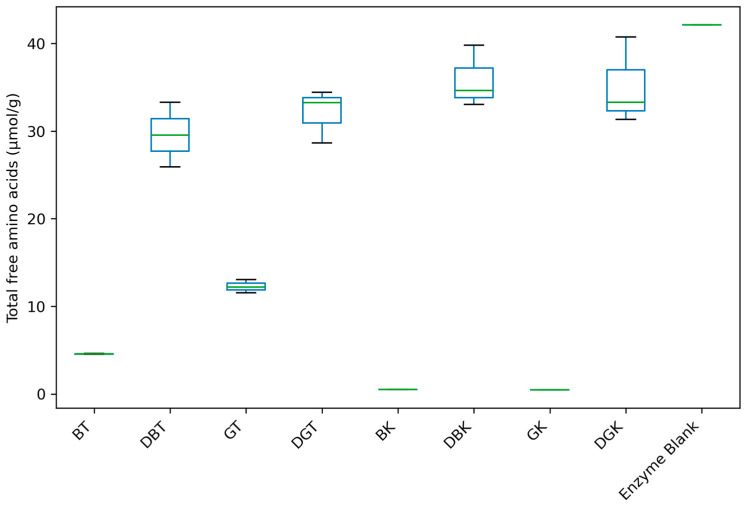
Total free amino acids’ content of black and green teas and kombuchas before and after in vitro gastrointestinal digestion.

**Figure 3:**
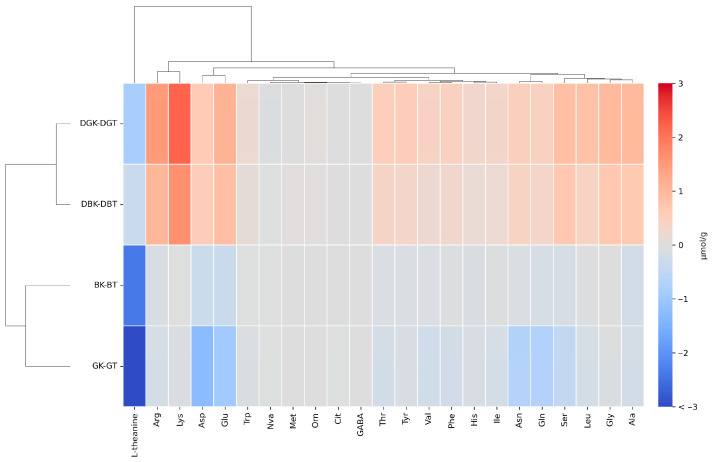
Comparison of the variation in the concentration of single amino acids, obtained by subtracting the values in tea from those in kombucha before and after in vitro gastrointestinal digestion through heatmap of hierarchical clustering. Asp (L-aspartic acid), Glu (L-glutamic acid), Asn (L-asparagine), Ser (L-serine), Gln (L-glutamine), His (L-histidine), Gly (Glycine), Thr (L-threonine), Cit (L-citrulline), Arg (L-arginine), Ala (L-alanine), GABA (gamma-aminobutyric acid), Tyr (L-tyrosine), Val (L-valine), Met (L-methionine), Ile (L-isoleucine), Phe (L-phenylalanine), Trp (L-tryptophan), Leu (L-leucine), Orn (L-ornithine), and Lys (L-lysine). L-norvaline (Nva): internal standard solution.

The fermentation and digestion processes can break down proteins and peptides into FAA, enhancing their bioaccessibility [69]. Therefore, a total of 22 amino acids were identified and quantified using derivatization with OPA (Figure 2 and Figure 3, Supplementary Table S2).

The quantified compounds included amino acids that are commonly found in tea and kombucha [70].

Total amino acids’ content (Figure 2) showed notable differences in amino acid concentration between non-digested tea and kombucha (8.43 ± 4.24 and 0.50 ± 0.03 µmol/g, respectively) and digested tea and kombucha (30.84 ± 3.33 and 35.48 ± 3.87 µmol/g, respectively) samples.

Among non-digested samples, GT showed the highest concentration (12.28 ± 0.75 µmol/g). In line with our findings, Jakubczyk et al. [71] reported a significantly higher total free amino acid content in GT compared to BT. Research showed that the overall reduction in free amino acid content during the processing of BT leaves was attributed to microbial extracellular enzymes, Maillard reactions, and the conversion of amino acids into volatile compounds [72,73].

The heatmap analysis (Figure 3) reports the variation in the concentration of individual amino acids, obtained by subtracting the values in tea from those in kombucha. In this way, we eliminate the interference caused by the degradation of the digestive enzyme used (enzyme blank).

The heatmap analysis reveals distinct clustering patterns among the various sample groups.

The undigested samples were very similar in free amino acids; concentration, except for L-theanine. L-theanine (γ-ethylamino-L-glutamic acid) is a highly water-soluble non-protein amino acid typically found in tea leaves and a glutamine derivative [74,75]. Moreover, it is known to be the most abundant amino acid in tea, constituting 50% of the total amino acids and 1% of the tea’s dry weight [76]. Glutamic acid and theanine are key contributors to the umami taste in green tea, enhancing its flavor profile. Following oral intake, it is efficiently and rapidly absorbed in the small intestine via sodium-dependent transporters located in the brush border membrane. Once in circulation, L-theanine can cross the blood–brain barrier and exert physiological effects in the central nervous system before being metabolized and excreted primarily through the urine [77]. Therefore, beyond taste, theanine is known for its calming effects on the mind. It promotes relaxation by influencing neurotransmitter activity, which helps lower blood pressure and reduce physiological responses to stress [78]. L-theanine was the most abundant amino acid in both BT (2.42 ± 0.02 µmol/g) and GT (5.97 ± 0.36 µmol/g). However, it showed a marked decrease in fermented samples, with a concentration of 0.04 ± 0.00 µmol/g in BK and was not detected in GK. This result aligns with the findings by Zhao et al. [79], who hypothesized that the reduction in theanine in kombucha may result from its interaction with the reducing substances produced during fermentation.

With the digestion process, L-theanine had a substantial decrease in both tea (0.33 ± 0.04 µmol/g in DBT and 0.83 ± 0.02 µmol/g in DGT) and kombucha samples (not detected). L-theanine is stable under acidic gastric conditions and resistant to heat degradation [80]. Its stability was documented across a pH range of 3.0 to 6.6, and no degradation was observed even after exposure to 121 °C for several minutes. The base-catalyzed hydrolysis of L-theanine into glutamic acid and ethylamine can occur under strongly alkaline conditions, typically found in the small intestinal environment (pH 7.5–8.5); this explains the strong degradation measured in the samples. Although, the high solubility, the fast assimilation, and the rapid absorption of L-theanine in this region counteract the significant chemical breakdown [77]. Furthermore, digestive enzymes in the small intestine do not specifically degrade L-theanine. On the basis of our results, we can suppose that theanine properties, registered in in vitro experiments, are only partially exerted in vivo due to theanine breakdown induced by intestinal digestion [81,82].

A slight decrease in several amino acids, mainly Glu and Asp in green tea, was detected after fermentation in the undigested samples. The observed differences in FAA concentrations in kombucha can be attributed to several factors, including the fermentation process, the type of tea used, and the microbial community involved in fermentation. Similarly, Zou et al. [83] observed a rapid decrease in FAA during the fermentation of green and black teas, with concentrations remaining at very low levels (<0.01 mg/mL). They explained that, since no supplemental nitrogen source was added during kombucha fermentation, the amino acids present in the sugared tea infusion likely became the primary nitrogen source for microbial growth, contributing to the reduction in FAA over time. This indicates that amino acids are being consumed by the fermenting microorganisms, possibly for cell growth and metabolic processes.

In the digested samples is evidence of the effect of microbial fermentation, as most amino acids showed a slight concentration increase in both kombuchas after digestion. The difference observed among the samples after digestion is related to the presence of microorganisms during the fermentation of tea, meaning that microorganisms in the kombucha release proteins and peptides that are digested and transformed into amino acids.

Among the amino acids measured, the main variations were related to Lys, Arg, Glu, Ser, Leu, Gly, and Ala. Among these amino acids, Lys and Glu are the most relevant for the yeast cell nitrogen composition, followed by Leu and Ala, although the aminoacidic protein composition in yeast cells depends on the growth conditions [84,85]. Moreover, among the most abundant, two essential amino acids were detected, Lys and Leu. The variation in the concentration of the former was the highest in digested kombuchas, and Lys was one of the main components of the SCOBY protein (53.1 mg/g of dry weight) [86]. This evidence suggests that the origin of these amino acids is related mainly to the digestion of microbial proteins released during kombuchas’ production, although the hydrolysis of protein of plant origin may not be excluded.

### 3.3. Antioxidant Activity

The antioxidant activities of kombucha may contribute to its various claimed health benefits, such as preventing different diseases related to oxidative stress, including the reduction in inflammation and arthritis symptoms and neuro-degenerative diseases and improved immune function [12,87]. The antioxidant activity of tea is mainly driven by its polyphenols, which act alongside vitamins E, A, and C to enhance its overall protective effects against oxidative stress [71]. It is well documented that GT has higher antioxidant activity compared to BT, largely due to its higher concentration of catechins and other polyphenols [88]. However, upon ingestion, phenolic compounds encounter a dynamic biochemical environment, transitioning from the highly acidic gastric phase (pH ~2) to the neutral or mildly alkaline small intestine (pH 6–7.5) and finally to the colon, where they are extensively metabolized by gut microbiota. These physiological transitions deeply affect the chemical integrity, solubility, and overall bioavailability of phenolic compounds [89]. After complete in vitro simulated digestion, the content and antioxidant activity values of gallic acid decreased significantly compared to the initial values before digestion, with the reduction becoming progressively more pronounced throughout the successive digestive phases [90]. Gallic acid, caffeic acid, and chlorogenic acid are not stable at high pH values. In contrast, catechin and epigallocatechin are relatively stable and did not show major pH-induced degradation. The stability is related to the structure of the compounds. Those with adjacent phenolic OH groups on a single benzene ring (caffeic, chlorogenic, gallic acids) were more susceptible to pH changes, possibly due to the formation of unstable quinone intermediates [91]. In ex vivo ileostomy fluid models [2], the green tea catechin (C), epicatechin (EC), epicatechin gallate (ECG), epigallocatechin (EGC), epigallocatechin gallate (EGCG), and gallic acid were metabolized during incubation with human ileal fluid under anaerobic conditions. Cleavage of the ester bonds of the gallated compounds ECG and EGCG was observed, releasing the aglycones EC, EGC, and gallic acid. After 24 h, pyrogallol was detected as a degradation product of gallic acid. The study showed the degradation of tea catechins and the formation of specific metabolites like valerolactones before reaching the colon.

Janhavi and colleagues [92] evaluated the bioaccessibility and bioavailability of the peel and rind polyphenols of sour mangosteen fruits by in vitro gastrointestinal and in vivo mouse models. The peel and rind extracts were rich in polyphenols like epicatechin, catechin, gallic acid, and others, the same as the green and black teas. In the in vitro study, epicatechin showed high bioaccessibility in the peel extract across oral, gastric, and intestinal phases. In the rind extract, gallic acid and catechin were highly bioaccessible. The results suggest that the polyphenols, especially epicatechin from the peel and catechin from the rind, are stable during gastrointestinal digestion and are highly bioavailable in vivo. After oral ingestion, EGCG undergoes unfavorable metabolic changes in the gastrointestinal tract and liver, leading to low bioavailability and limited organ access. Factors include its polar nature, enzymatic conversion to inactive metabolites, efflux pumps, poor transcellular transport, and interactions with gut microbiota. In the GI tract, EGCG is inactivated [93].

**Figure 4:**
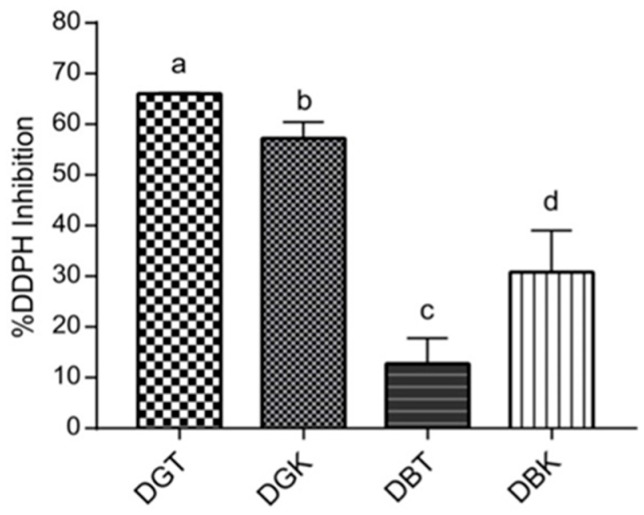
Antioxidant activity of digested samples (DBT, DBK, DGT, and DGK) expressed as % DPPH inhibition. Different letters indicate significant differences between values (*p* < 0.05).

The DPPH assay measures antioxidant activity through the reaction of antioxidants with nitrogen-centered radicals, which mimic peroxyl radical found in biological systems [94]. This assay evaluated the antioxidant capacity of tea and kombucha samples after simulated digestion to assess the antioxidants available for absorption. The DPPH radical scavenging activity of the four different digested samples was measured, and the results are shown in Figure 4.

The percentage of inhibition of DPPH radicals varied significantly among the samples, as indicated by different letters representing distinct groups (*p* < 0.05). DGT exhibited the highest antioxidant activity likely due to its higher phenolic content, which aids in radical scavenging and alleviating oxidative stress [94]. DGT demonstrated a DPPH inhibition percentage of 66.02 ± 0.16%, followed by DGK, which showed significant inhibition of 57.21 ± 2.64% (*p* < 0.05). Conversely, DBK displayed a higher inhibition rate (30.80 ± 6.71%) compared to DBT (12.75 ± 4.08%). When comparing the two types of tea leaves used in kombucha production (green and black), the digested green tea and kombucha (DGT and DGK) exhibited greater antioxidant activity after digestion than their black tea and kombucha counterparts (DBT and DBK). This difference is attributed to the unique composition of green leaves, as previously reported, which boosts the antioxidant properties of the digested samples compared to black leaves. The pronounced effect on antioxidant activity in digested kombucha samples was more evident in DBK, probably due to the greater influence of microbial fermentation on phenolic compounds. A slight difference in antioxidant activity between DGK and DGT may be attributed to variations in phenolic compounds resulting from fermentation, leading to different stabilities of phenols under digestion conditions [95]. The primary polyphenolic compounds, catechin and epicatechin, are well known for their potent antioxidant properties [96]. As reported by Chakravorty et al. [97] and de Noronha et al. [58], the fermentation process leads to an increase in polyphenol content. For example, during fermentation, epicatechin isomers can undergo hydrolysis, leading to increased levels of compounds like gallic acid, epicatechin, and epigallocatechin. These transformations not only boost the overall phenolic content but also significantly enhance the antioxidant activity of the final product. The variability in antioxidant potential highlights the influence of fermentation and digestion on the chemical composition of the samples. Approximately 48% of polyphenols are digested in the small intestine and 42% in the large intestine, suggesting that kombucha’s antioxidant components have varying stability against digestion [98]. A study investigating the antioxidant properties of gooseberries as affected by simulated gastrointestinal digestion found a significant increase in the antioxidant activity measured by DPPH assay [99]. A possible explanation for the higher DPPH inhibition in DBK compared to DBT is that during the fermentation of tea into kombucha many enzymes are produced, such as phytase, α-galactosidase, and tannase [96]. These microbial enzymes are involved in the oxidation of polyphenol structures, which leads to the formation of catechins, flavonoids, and other compounds with radical scavenging properties [27,57]. This microbial hydrolysis reaction subsequently increases the total phenolic compounds [96,100,101].

Conversely, a slight difference in antioxidant activity between DGK and DGT may be attributed to variations in phenolic compounds resulting from fermentation, leading to different stabilities of phenols under digestion conditions [95]. In particular, pH can influence biological reactivity by affecting the racemization of molecules [102,103]. Consequently, different chiral enantiomers exhibit varying reactivities with respective reagents. This could result in kombucha antioxidants being more reactive early in the digestive process, especially at acidic pH during the gastric phase, and less reactive at higher pH after the intestinal phase [35,101]. This study reveals the significant variability in antioxidant activity among digested kombucha and tea samples, demonstrating the importance of fermentation and digestion processes in determining their phenolic composition and stability.

### 3.4. Antimicrobial Activity

The use of natural antimicrobial compounds recently gained significant attention, driven by consumer demand for minimally processed foods that retain their nutritional value and safety [104]. These natural agents offer a promising alternative to synthetic preservatives, addressing health concerns and enhancing the overall quality and shelf life of food products [104]. Recent studies highlighted kombucha’s potential to modulate gut microbiota and inhibit pathogenic bacteria [105,106,107]. However, the retention of its antimicrobial properties and bioactive compounds after digestion remains underexplored [108]. Evaluating these compounds after complete in vitro digestion is important for assessing their role in food safety and gut health. *S. enterica* subsp. *enterica* serovar Typhimurium SL1344 is widely used in research due to its well-characterized virulence. It causes gastroenteritis and can invade intestinal epithelial cells, leading to systemic infections [109]. *Y. enterocolitica* Xen24 is known for causing yersiniosis, which manifests as gastrointestinal disturbances and is often found in contaminated food, particularly pork [110]. *S. sonnei* ATCC 11060 is a significant cause of bacillary dysentery, while *L. monocytogenes* EGD-e (ATCC BAA-679) causes listeriosis, especially in immunocompromised individuals [111]. The strain EGD-e is extensively studied for its ability to invade and replicate within host cells [112]. The enterotoxigenic (ETEC) *E. coli* strain is associated with infant diarrhea in developing countries. In specific conditions, it produces heat-labile and/or heat-stable enterotoxins that disrupt the normal fluid balance in the intestines [113,114].

**Figure 5:**
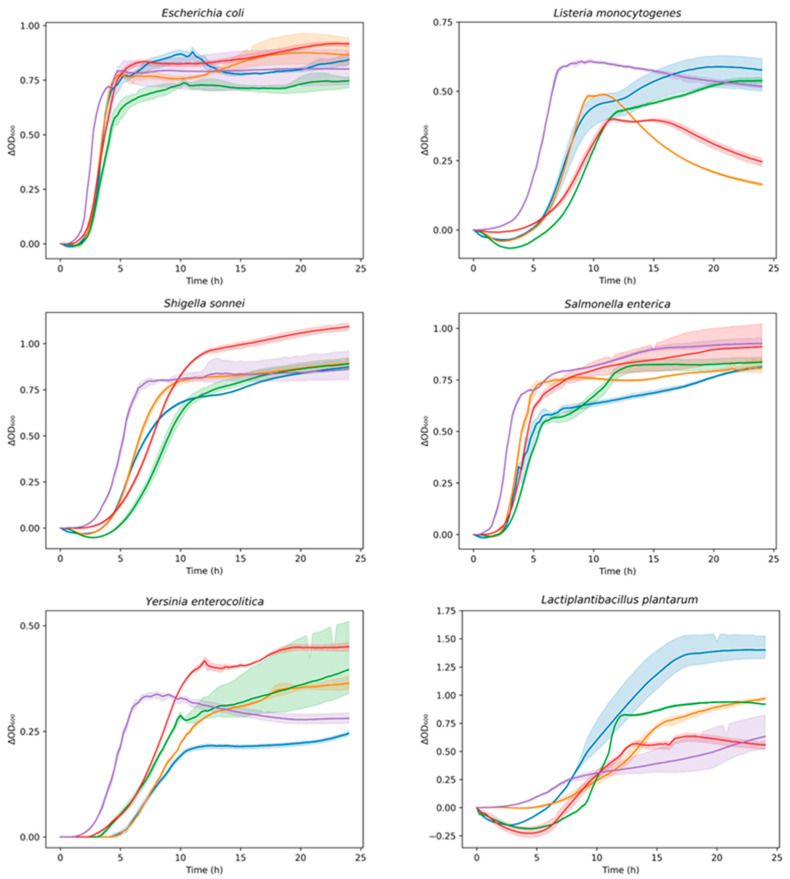
Growth curves of indicator bacterial strains in presence of digested samples (DBT, DBK, DGT, and DGK) over time (24 h). Data are expressed as optical density (ΔOD_600_) as a function of time. DBK (▬), DGK (▬), DBT (▬), DGT (▬), Control (▬). The shadow surrounding the curves (mean) represents the SD of replicates. Control: indicator strain without digested sample.

The antimicrobial activity of digested tea and kombucha samples on the growth curve of different indicator strains is reported in Figure 5.

**Figure 6:**
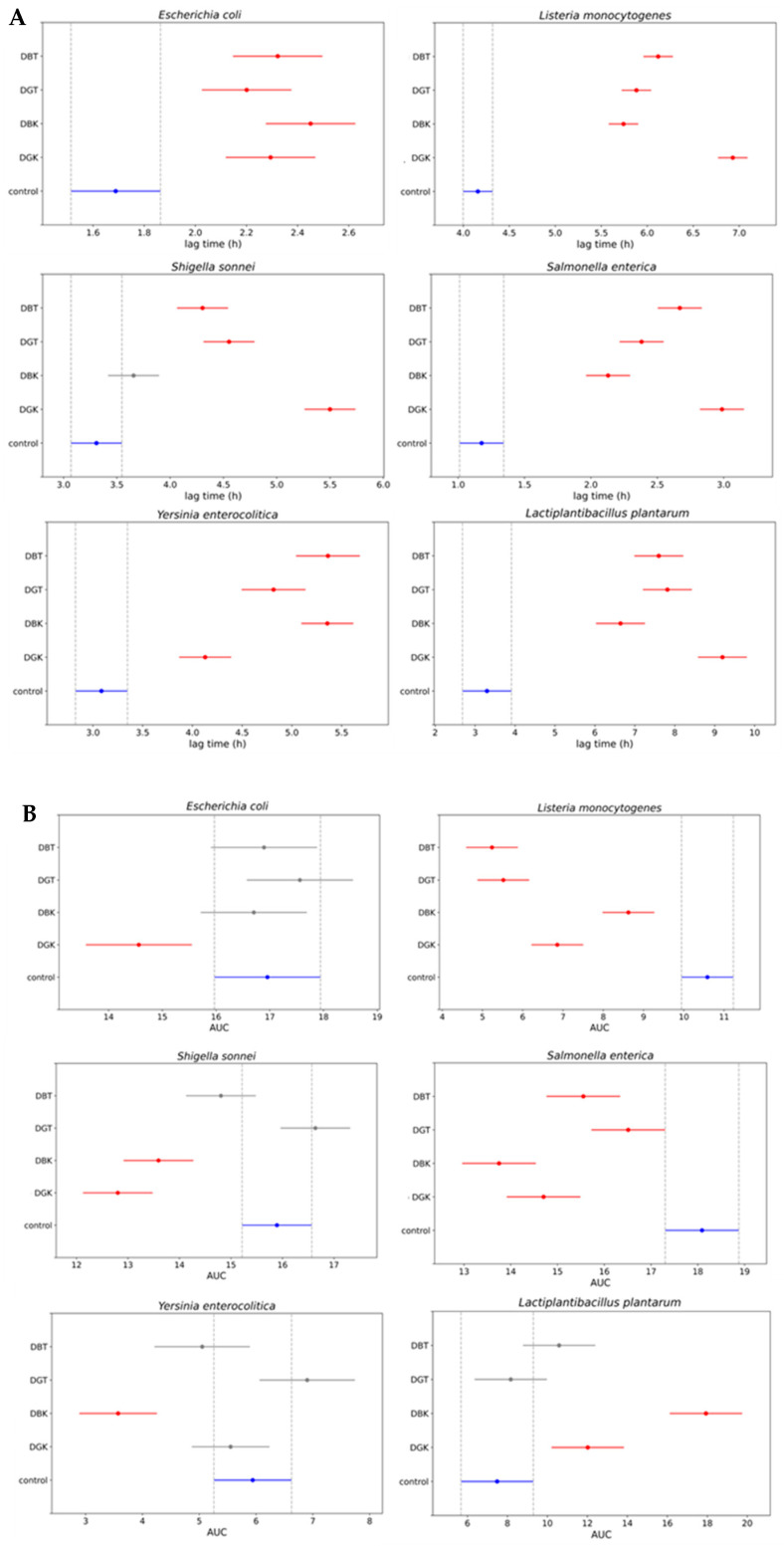
Comparison of the growth of indicator bacterial strains in presence of digested black tea (DBT), digested green tea (DGT), digested black kombucha (DBK), and digested green kombucha (DGK). Simultaneous control graph of 95% confidence intervals from Tukey’s HSD of (**A**) the area under the curve (AUC) values and (**B**) the lag phase duration time values. Non-overlapping bars indicate a statistically significant difference at *p* < 0.05, whereas overlapping bars indicate no significant difference. Red bars indicate samples that differ significantly from the control (blue bar), while gray bars indicate samples that do not differ significantly from the control. Control: indicator strain without digested sample.

To better quantify the inhibition and stimulation effects, the area under the growth curve (AUC) was calculated (Figure 6A).

This provided a numerical quantification of the differences in growth among the various combinations. The AUC average values (95% confidence interval) of digested samples were compared to those of the positive control (indicator strain without digested sample). A lower AUC value compared to positive control represented the inhibition of indicator strain growth in the presence of a digested sample. The digested kombucha samples (DBK and DGK) demonstrated inhibition of *L. monocytogenes*, *S. sonnei*, and *S. enterica*. In particular, DBK exhibited the highest inhibition against *S. enterica* compared to other samples and was the only one effective against *Y. enterocolitica*. A reduction in growth was observed for the *E. coli* strain only in the presence of DGK. Regarding the LAB inserted as a representative of the gut microbiota (*L. plantarum*), the impact varied significantly among the samples. As indicated by the AUC values for the digested samples in combination with *L. plantarum*, a statistically significant increase in the growth of the LAB was observed for the digested kombucha samples (DBK and DGK). In contrast, this effect was not significant for the digested tea samples (DBT and DGT). This highlights the potential of digested kombucha in modulating microbial growth after digestion, with variations based on the bacterial species and sample type. Digested kombucha samples were found to have no inhibitory effect against *L. plantarum*. This could be due to phenolic compounds that act similarly to prebiotics, reaching the large intestine where they serve as substrates for beneficial gut bacteria [115]. In turn, these bacteria enhance polyphenol bioaccessibility by breaking them down into smaller molecules [24,116].

To analyze the inhibition dynamics in more detail, the lag phase from the bacterial growth curves was calculated using the Gompertz equation [55] (Figure 6B). The modeling with the modified Gompertz equation demonstrated a strong correlation, as indicated by the mean R-squared value of 0.986 ± 0.016. The lag phase duration was used as a key indicator to assess inhibitory effects, with longer lag times reflecting a delay in microbial adaptation and initiation of growth.

The *E. coli* strain exhibited a delay in the lag phase when in combination with all the digested samples. As shown by the AUC values for *L. monocytogenes*, all samples demonstrated growth inhibition, with DGK displaying the most substantial increases in lag time (~7 h), indicating strong antimicrobial properties. For *S. sonnei*, all treated groups, except DBK, exhibited significantly longer lag times compared to the control, with DGK showing the greatest inhibition (~5.5 h). Similarly, DGK was particularly effective against *S. enterica*, prolonging the lag phase by approximately 3 h. The AUC values of samples in combination with *Y. enterocolitica* indicated antimicrobial activity only for DBK, although all samples prolonged the lag phase. *L. plantarum* exhibited increased lag times in all treatment groups. Despite the prolonged lag phase, which suggests slower initial adaptation, the overall growth (AUC) in the presence of the digested kombucha samples was greater than that of the control. Different dynamics observed with the lag phase calculated by the modified Gompertz equation demonstrated a slightly different behavior compared to AUC values.

Nowadays, drug-resistant pathogenic bacteria pose a major challenge to human health and the pharmaceutical industry. Kombucha tea has been studied by many researchers for its inhibitory activity on many pathogenic microorganisms. The antimicrobial activity of kombucha tea is largely attributable to the presence of organic acids (acetic acid, gluconic acid, glucuronic acid, etc.), bacteriocins, enzymes, tea polyphenols, and their derivatives [60,117,118]. Organic acids, particularly acetic acid, are among the most well-documented antimicrobial agents in kombucha. Acetic acid lowers the pH of the medium, creating an unfriendly environment for many pathogenic microorganisms [119,120]. Other organic acids, such as gluconic acid, also contribute to microbial inhibition [121]. Polyphenols that originate primarily from the tea also play a central role in antimicrobial activity. Polyphenols exhibit antimicrobial activity by disrupting microbial growth through interactions with cell membranes, enzyme activities, and essential cellular processes [122]. During fermentation, the amount of polyphenols increases. Moreover, catechins present in the tea may be degraded by bacteria and yeast to simpler particles, thus increasing the antioxidative strength [57]. Epigallocatechin, epicatechin gallate, and epigallocatechin gallate were found to be inhibitory for the growth of *S. aureus* and *V. cholerae* [123]. Bacteriocins (bioactive peptides that have an antimicrobial effect on other species) were identified in kombucha [3,124] and can contribute to its antimicrobial activity. A recent study by Pei and colleagues [124] purified and characterized a bacteriocin produced by an *L. plantarum* strain isolated from kombucha. This novel bacteriocin was active against both Gram-positive and Gram-negative bacteria and acts by increasing the permeability of the cell membrane, which eventually leads to bacterial cell death. Moreover, it can also inhibit *S. aureus* biofilm formation. Prolonged storage at 37 °C and treatment with the stomach proteases pepsin and trypsin did not affect its antibacterial properties, suggesting stability in the human body. Together, organic acids, polyphenols, and bacteriocins act synergistically to inhibit a broad range of microbial pathogens, contributing to the growing interest in kombucha as a natural antimicrobial agent and food preservative.

Additionally, specific proteins and exopolysaccharides secreted during food fermentation may inhibit pathogen adhesion to the intestinal mucosa [23].

The AUC values reported in Figure 6A demonstrated varying inhibition dynamics in the growth of the tested indicator strains. DGK demonstrated significant inhibition of the growth of the tested Gram-positive indicator *L. monocytogenes*. This could be due to the presence of antimicrobial components in kombucha, other than organic acids [119,121], that remain effective even after digestion. In fact, one of the main antimicrobial compounds present in kombucha is acetic acid, whose effect is neutralized by the normalization of pH samples during the intestinal phase to value 7. In addition, another reason for the differences in bacterial susceptibility could be probably the complex structure of the outer membrane surrounding the cell wall in the Gram-negative bacteria tested, such as *E. coli*, *S. sonnei*, *S. enterica*, and *Y. enterocolitica* [118]. This outer protective envelope restricts the diffusion of compounds through its lipopolysaccharide covering, as previously reported by Breijyeh et al. [125] and Battikh et al. [126]. The antimicrobial activity observed after digestion indicates that bioactive compounds remain effective in the gastrointestinal tract, providing protection against pathogens.

### 3.5. Culture-Dependent Microbial Viability

**Figure 7:**
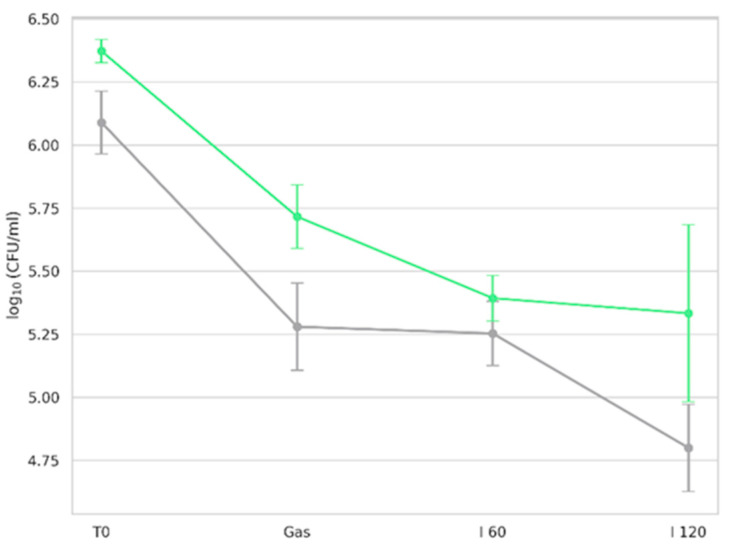
Viability of kombucha microorganisms during in vitro digestion at different phases, assessed on plate count agar. T0 (initial microbial count before digestion); Gas (after gastric phase); I 60 (after 60 min of intestinal phase); I 120 (at the end of digestion, 120 min). Green line represents counts for green kombucha, while gray line represents counts for black kombucha. Results are expressed as mean ± SD.

The viability of microorganisms in GK and BK during simulated gastrointestinal digestion is shown in Figure 7.

Viability was expressed as log CFU/mL based on enumeration on the PCA culture medium. No microbial growth was observed in enzyme blank controls, confirming the absence of contamination from digestion reagents. Before exposure to digestive fluids, both kombucha types exhibited robust microbial populations. Following the gastric phase, comprising 1:1 (*v*/*v*) dilution of the oral bolus with simulated gastric fluid (SGF), including pepsin at pH 3.0 under agitation for 2 h at 37 °C [39], a notable reduction in microbial counts was observed. In a recent study, Wang et al. [127] demonstrated that yeast strains isolated from commercial kombucha showed high tolerance to low pH, especially at pH 3. Similarly, AAB of the genera *Komagataeibacter*, *Acetobacter*, and *Gluconobacter* displayed sustained growth at pH 3 [7]. Such acid tolerance is a well-established trait of microbes isolated from fermented products. However, the gastric environment includes not only low pH but also proteolytic activity. Pepsin, a key gastric enzyme, degrades proteins including membrane-associated proteins of microbial cells. This dual stressor (low pH + pepsin) contributed to the reduction in viable counts. Despite reductions in our study, viable counts remained above 5 log CFU/mL post-gastric digestion, suggesting resilience of the kombucha microbiota under gastric-like conditions.

Following gastric digestion, samples were exposed to simulated intestinal fluid (SIF), with viability assessed at 60 min (I 60) and 120 min (I 120). The decline in viability during the intestinal phase is attributable to bile salt activity. Bile used in this study (10 mM, bovine origin) mimicked human bile composition and concentration (0.6%) [128]. Bile salts, being amphiphilic, disrupt bacterial membranes and are known to inhibit microbial survival in the intestine [129]. Sharifudin et al. [130] also observed that, among kombucha isolates, only *D. bruxellensis* and *L. plantarum* tolerated 4 h of exposure to 0.3–1% bile salts. Despite the stress of bile exposure, both GK and BK maintained viable counts of ~5 log CFU/mL after 2 h of gastric and 2 h of intestinal digestion, supporting the potential for delivering live microorganisms to the gut following consumption.

### 3.6. Culture-Independent Microbial Viability by PMA-Based DNA Sequencing

**Figure 8:**
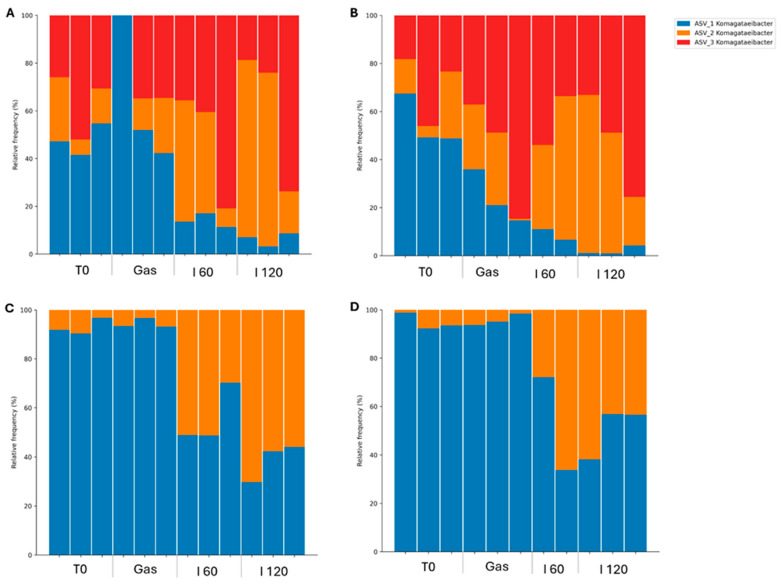
Relative abundance of *Komagataeibacter* amplicon sequence variants across various stages of gastrointestinal digestion. (**A**) Green kombucha; (**B**) green kombucha treated with PMAxx; (**C**) black kombucha; (**D**) black kombucha treated with PMAxx. PMAxx treatment ensured that the DNA originated exclusively from live cells.

Metabarcoding analyses were performed to evaluate the relative abundance of viable bacterial and fungal taxa in GK and BK during in vitro simulated digestion. PMAxx-treated samples enabled the selective amplification of DNA from live cells by excluding DNA from dead or membrane-compromised cells. However, amplification failed for one replica of PMA-treated GK samples after the gastric phase and one replica of BK samples after 60 min of intestinal digestion, due to insufficient DNA yields detected during post-extraction quantification. The kombucha microbiota are highly variable, influenced by factors such as the composition of starter cultures, tea type, sugar concentration, fermentation time, and temperature [9]. In this study, the microbial community structure was characterized by sequencing the V3–V4 region of the 16S rRNA gene for bacteria and the ITS1 region for fungi. Control samples containing only the enzyme mixtures were also sequenced to identify and eliminate potential reagent-derived contaminants. After quality filtering, contaminant removal, and applying the abundance threshold, only one bacterial genus, *Komagataeibacter,* was consistently detected across samples. Similarly, fungal sequencing revealed a single dominant taxon: *Saccharomyces* spp., represented by one amplicon sequence variant (ASV) with over 90% relative abundance. Due to the low diversity observed, yeast data were excluded from further discussion. Figure 8 displays the relative abundance of four *Komagataeibacter* ASVs across digestion stages: before digestion (T0), after gastric exposure (Gas), and after 60 and 120 min in simulated intestinal fluid (I 60 and I 120) for both GK (A, B) and BK (C, D).

*Komagataeibacter* is widely recognized as the dominant bacterial genus in kombucha, commonly found in both the SCOBY and the beverage itself [8,131]. It includes species such as *K. xylinus*, *K. europaeus*, *K. hansenii*, *K. rhaeticus*, and *K. medellinensis* [132,133]. The genus was reclassified from *Gluconacetobacter* based on phylogenetic updates [134]. At baseline (T0), GK was dominated by ASVs 1 and 3, while BK exhibited high levels of ASV 1 with lower contributions from ASV 2. Despite being produced from the same SCOBY, the kombuchas displayed distinct ASV profiles, consistent with reports that different tea substrates shape microbial communities [135]. ASVs 1 and 2 were shared between both kombuchas, while ASV 3 was exclusive to GK. After gastric digestion, GK showed a modest decline in ASV 1, accompanied by a slight increase in other ASVs. In contrast, BK maintained a stable *Komagataeibacter* profile during this phase. However, in the intestinal phase, a marked reduction in ASV 1 was observed in both kombuchas. In BK, ASV 1 declined by approximately 50%. In GK, ASV 1 remained detectable after 2 h of SIF exposure but at very low levels, suggesting differential susceptibility to intestinal conditions. ASV 2 demonstrated higher resilience, persisting in both beverages throughout digestion, making it the most promising candidate in terms of viability. These differences suggest that certain *Komagataeibacter* variants may possess greater tolerance to digestive stressors. While AAB have not been widely established as probiotics, emerging studies highlight their potential due to their fermentation activity, acid resistance, and possible health-promoting effects [7,136]. Nevertheless, their probiotic characterization remains unexplored. According to current probiotic criteria, microorganisms intended for functional food use must tolerate gastrointestinal conditions [137,138,139]. In this context, our PMA-based approach offers preliminary evidence that certain *Komagataeibacter* strains can survive conditions mimicking gastrointestinal passage. Although both kombuchas maintained microbial counts around 5 log CFU/mL following simulated gastrointestinal digestion, this level may still fall below the threshold generally considered necessary to achieve probiotic effects. Therefore, while these findings confirm the resilience of some microorganisms, further work is needed to isolate these strains and characterize their probiotic potential. If kombucha is to be developed as a live microbe or probiotic-based beverage, fermentation conditions should be optimized to achieve initial cell concentrations exceeding 7 log CFU/mL. This would help ensure that, even after exposure to gastrointestinal stress, a sufficient number of viable cells remain available to potentially confer health benefits.

## 4. Conclusions

This study underscores the impact of both fermentation and gastrointestinal digestion on the functional properties of green and black teas. Results demonstrate that, although the free *N*-terminal content was very similar between tea and kombucha, digestion significantly increased this content in fermented products compared to unfermented tea. This suggests that microbial fermentation facilitates the release of proteins and peptides suitable for gastrointestinal digestion. Digestion also altered the free amino acid profiles, though with a similar overall pattern for both matrices. Among the positive effects of fermentation, higher antioxidant activity was observed in BK compared to BT after digestion. Moreover, both digested kombuchas showed enhanced antimicrobial activity against several pathogens and promoted the growth of *L. plantarum*, a common gut commensal. In addition to chemical and functional changes, microbial viability analysis confirmed that both kombucha types retained viable microbial populations after digestion, with *Komagataeibacter* strains, particularly one resilient ASV, surviving simulated gastrointestinal conditions. These findings suggest that kombucha may serve not only as a source of bioactive compounds but also as a vehicle for potentially probiotic microorganisms. However, in order to be classified as probiotics, these microorganisms must meet all established criteria, including safety, functional efficacy, and survival through the gastrointestinal tract. Furthermore, fermentation conditions may need to be optimized to achieve higher initial microbial loads, ensuring that a sufficient number of viable cells remain available post-digestion to potentially confer health benefits. Overall, this study demonstrates that the functional properties of kombucha are maintained or enhanced after gastrointestinal digestion, supporting its health-promoting potential. Future research should focus on elucidating the mechanisms underlying these effects, especially the bioavailability of bioactive compounds and the role of viable microbes in gut health and well-being.

## Figures and Tables

**Table 1:** Composition of tea (BT and GT) and kombucha (BK and GK) samples.

Sample	BT	BK	GT	GK
pH	4.95 ± 0.04 ^b^	3.00 ± 0.12 ^c^	5.31 ± 0.04 ^a^	3.17 ± 0.06 ^c^
Moisture (g/100 mL)	94.47 ± 0.05 ^c^	95.70 ± 0.08 ^b^	94.71 ± 0.05 ^c^	96.89 ± 0.06 ^a^
Dry matter (g/100 mL)	5.53 ± 0.07 ^a^	4.27 ± 0.08 ^b^	5.28 ± 0.06 ^a^	3.11 ± 0.06 ^c^
Ash (g/100 mL)	0.05 ± 0.03 ^a^	0.01 ± 0.02 ^a^	0.02 ± 0.01 ^a^	0.03 ± 0.01 ^a^
Crude protein (g/100 mL)	0.08 ± 0.02 ^a^	0.06 ± 0.01 ^a^	0.06 ± 0.01 ^a^	0.07 ± 0.01 ^a^
Sucrose (g/L)	55.80 ± 0.30 ^a^	22.43 ± 0.40 ^b^	57.09 ± 1.24 ^a^	12.35 ± 0.38 ^c^
D-glucose (g/L)	ND	1.81 ± 0.01 ^b^	ND	3.67 ± 0.05 ^a^
Fructose (g/L)	ND	4.74 ± 0.04 ^b^	ND	9.39 ± 0.17 ^a^

Values are mean ± SD from three independent measurements. Values within the same row that do not share the same letter are significantly different from each other (*p* < 0.05).

## Data Availability

The original contributions presented in the study are included in the article/Supplementary Materials. Further inquiries can be directed to the corresponding author.

## Supplementary Materials

The following supporting information can be downloaded at https://www.mdpi.com/article/10.3390/foods14162770/s1. Table S1: Growth curves’ raw data of indicator bacterial strains in presence of digested samples (DBT, DBK, DGT, and DGK) over time (24 h). Data are expressed as optical density (OD_600_). Table S2: Concentrations of amino acids (µmol/g) across different sample groups (GT, GK, BT, BK, DGK, DBK, DGT, DBT). “ND” indicates that the compound was not detected in the respective sample group.

## References

[1] Hilal Y., Engelhardt U. (2007). Characterisation of white tea—Comparison to green and black tea. J. Verbraucherschutz Leb..

[2] Schantz M., Erk T., Richling E. (2010). Metabolism of green tea catechins by the human small intestine. Biotech. J..

[3] Bogdan M., Justine S., Filofteia D.C., Petruța C.C., Gabriela L., Roxana U.E., Florentina M. (2018). Lactic acid bacteria strains isolated from kombucha with potential probiotic effect. Rom. Biotechnol. Lett..

[4] Grassi A., Cristani C., Palla M., Di Giorgi R., Giovannetti M., Agnolucci M. (2022). Storage time and temperature affect microbial dynamics of yeasts and acetic acid bacteria in a kombucha beverage. Int. J. Food Microbiol..

[5] Ribič A., Trček J. (2024). Customized 16S-23S rDNA ITS Amplicon Metagenomics for Acetic Acid Bacteria Species Identification in Vinegars and Kombuchas. Microorganisms.

[6] Tran T., Grandvalet C., Verdier F., Martin A., Alexandre H., Tourdot-Maréchal R. (2020). Microbial Dynamics between Yeasts and Acetic Acid Bacteria in Kombucha: Impacts on the Chemical Composition of the Beverage. Foods.

[7] Wang B., Rutherfurd-Markwick K., Liu N., Zhang X.-X., Mutukumira A.N. (2024). Probiotic potential of acetic acid bacteria isolated from kombucha in New Zealand in vitro. Microbe.

[8] Gaggìa F., Baffoni L., Galiano M., Nielsen D.S., Jakobsen R.R., Castro-Mejía J.L., Bosi S., Truzzi F., Musumeci F., Dinelli G. (2019). Kombucha beverage from green, black and rooibos teas: A comparative study looking at microbiology, chemistry and antioxidant activity. Nutrients.

[9] Içen H., Corbo M.R., Sinigaglia M., Korkmaz B.I.O., Bevilacqua A. (2023). Microbiology and antimicrobial effects of kombucha, a short overview. Food Biosci..

[10] Han D., Yang Y., Guo Z., Dai S., Jiang M., Zhu Y., Wang Y., Yu Z., Wang K., Rong C. (2024). A Review on the Interaction of Acetic Acid Bacteria and Microbes in Food Fermentation: A Microbial Ecology Perspective. Foods.

[11] Kruk M., Trząskowska M., Ścibisz I., Pokorski P. (2021). Application of the “scoby” and kombucha tea for the production of fermented milk drinks. Microorganisms.

[12] Leal J.M., Suárez L.V., Jayabalan R., Oros J.H., Escalante-Aburto A. (2018). A review on health benefits of kombucha nutritional compounds and metabolites. CYTA-J. Food.

[13] Aung T., Eun J.B. (2022). Impact of time and temperature on the physicochemical, microbiological, and nutraceutical properties of laver kombucha (*Porphyra dentata*) during fermentation. Lwt.

[14] Cardoso R.R., Moreira L.D.P.D., De Campos Costa M.A., Toledo R.C.L., Grancieri M., Do Nascimento T.P., Ferreira M.S.L., Da Matta S.L.P., Eller M.R., Duarte Martino H.S. (2021). Kombuchas from green and black teas reduce oxidative stress, liver steatosis and inflammation, and improve glucose metabolism in Wistar rats fed a high-fat high-fructose diet. Food Funct..

[15] Fraiz G.M., Costa M.A.C., Cardoso R.R., James R.H., Zhao L., Corich V., Giacomini A., Milagro I., Barros F.A.R., Bressan J. (2024). Black Tea Kombucha Consumption: Effect on Cardiometabolic Parameters and Diet Quality of Individuals with and without Obesity. Fermentation.

[16] Villarreal-Soto S.A., Beaufort S., Bouajila J., Souchard J.P., Renard T., Rollan S., Taillandier P. (2019). Impact of fermentation conditions on the production of bioactive compounds with anticancer, anti-inflammatory and antioxidant properties in kombucha tea extracts. Process Biochem..

[17] Batista P., Rodrigues Penas M., Vila-Real C., Pintado M., Oliveira-Silva P. (2023). Kombucha: Challenges for health and mental health. Foods.

[18] Coelho R.M.D., de Almeida A.L., do Amaral R.Q.G., da Mota R.N., de Sousa P.H.M. (2020). Kombucha: Review. Int. J. Gastron. Food Sci..

[19] Fu C., Yan F., Cao Z., Xie F., Lin J. (2014). Antioxidant activities of kombucha prepared from three different substrates and changes in content of probiotics during storage. Food Sci. Technol..

[20] Kozyrovska N.O., Reva O.M., Goginyan V.B., Devera J.P. (2012). Kombucha microbiome as a probiotic: A view from the perspective of post-genomics and synthetic ecology. Biopolym. Cell..

[21] Kim J., Adhikari K. (2020). Current trends in kombucha: Marketing perspectives and the need for improved sensory research. Beverages.

[22] Harrison K., Navarro R., Jensen K., Cayler W., Nielsen T., Curtin C. (2023). Live, probiotic, or neither? Microbial composition of retail-available kombucha and “hard” kombucha in the Pacific Northwest of the United States. Beverages.

[23] Marco M.L., Heeney D., Binda S., Cifelli C.J., Cotter P.D., Foligné B., Gänzle M., Kort R., Pasin G., Pihlanto A. (2017). Health benefits of fermented foods: Microbiota and beyond. Curr. Opin. Biotechnol..

[24] Vargas B.K., Fabricio M.F., Záchia Ayub M.A. (2021). Health effects and probiotic and prebiotic potential of kombucha: A bibliometric and systematic review. Food Biosci..

[25] Dahiya Nigham Dahiya D., Nigam P.S. (2022). Probiotics, prebiotics, synbiotics, and fermented foods as potential biotics in nutrition improving health via the microbiome-gut-brain axis. Fermentation.

[26] Değirmencioğlu N., Yıldız E., Sahan Y., Güldas M., Gürbüz O. (2021). Impact of tea leaves types on antioxidant properties and bioaccessibility of kombucha. J. Food Sci. Technol..

[27] Tamer C.E., Temel S.G., Suna S., Ozkan Karabacak A., Ozcan T., Yilmaz Ersan L., Turkol Kaya B., Copur O.U. (2021). Evaluation of bioaccessibility and functional properties of kombucha beverages fortified with different medicinal plant extracts. Turkish J. Agric. For..

[28] Vitas J.S., Popović L.M., Čakarević J.C., Malbaša R.V., Vukmanović S.Z. (2020). In Vitro Assessment of Bioaccessibility of the Antioxidant Activity of Kombucha Beverages After Gastric and Intestinal Digestion. Food Feed Res..

[29] Alevia M., Rasines S., Cantero L., Teresa Sancho M., Fernández-Muiño M.A., Osés S.M. (2021). Chemical extraction and gastrointestinal digestion of honey: Influence on its antioxidant, antimicrobial and anti-inflammatory activities. Foods.

[30] Lorieau L., Halabi A., Ligneul A., Hazart E., Dupont D., Floury J. (2018). Impact of the dairy product structure and protein nature on the proteolysis and amino acid bioaccessiblity during in vitro digestion. Food Hydrocoll..

[31] Majdoub Y.O.E., Ginestra G., Mandalari G., Dugo P., Mondello L., Cacciola F. (2021). The digestibility of *Hibiscus sabdariffa* L. Polyphenols using an in vitro human digestion model and evaluation of their antimicrobial activity. Nutrients.

[32] Piscopo M., Tenore G.C., Notariale R., Maresca V., Maisto M., de Ruberto F., Heydari M., Sorbo S., Basile A. (2020). Antimicrobial and antioxidant activity of proteins from Feijoa sellowiana Berg. fruit before and after in vitro gastrointestinal digestion. Nat. Prod. Res..

[33] Sánchez-Gutiérrez M., Gómez-García R., Carrasco E., Bascón-Villegas I., Rodríguez A., Pintado M. (2022). Quercus ilex leaf as a functional ingredient: Polyphenolic profile and antioxidant activity throughout simulated gastrointestinal digestion and antimicrobial activity. J. Funct. Foods.

[34] Seraglio S.K.T., Valese A.C., Daguer H., Bergamo G., Azevedo M.S., Nehring P., Gonzaga L.V., Fett R., Costa A.C.O. (2017). Effect of in vitro gastrointestinal digestion on the bioaccessibility of phenolic compounds, minerals, and antioxidant capacity of Mimosa scabrella Bentham honeydew honeys. Food Res. Int..

[35] Wootton-Beard P.C., Moran A., Ryan L. (2011). Stability of the total antioxidant capacity and total polyphenol content of 23 commercially available vegetable juices before and after in vitro digestion measured by FRAP, DPPH, ABTS and Folin-Ciocalteu methods. Food Res. Int..

[36] Cardoso R.R., Neto R.O., dos Santos D’Almeida C.T., do Nascimento T.P., Pressete C.G., Azevedo L., Martino H.S.D., Cameron L.C., Ferreira M.S.L., de Barros F.A.R. (2020). Kombuchas from green and black teas have different phenolic profile, which impacts their antioxidant capacities, antibacterial and antiproliferative activities. Food Res. Int..

[37] Association of Official Analytical Chemists (AOAC) (2016). Official Methods of Analysis of the AOAC International.

[38] Association of Official Analytical Chemists (AOAC) (2005). Official Methods of Analysis of the AOAC International.

[39] Brodkorb A., Egger L., Alminger M., Alvito P., Assunção R., Ballance S., Bohn T., Bourlieu-Lacanal C., Boutrou R., Carrière F. (2019). INFOGEST static in vitro simulation of gastrointestinal food digestion. Nat. Protoc..

[40] Egger L., Ménard O., Delgado-Andrade C., Alvito P., Assunção R., Balance S., Barberá R., Brodkorb A., Cattenoz T., Clemente A. (2016). The harmonized INFOGEST in vitro digestion method: From knowledge to action. Food Res. Int..

[41] Tanambell H., Danielsen M., Devold T.G., Møller A.H., Dalsgaard T.K. (2024). In vitro protein digestibility of RuBisCO from alfalfa obtained from different processing histories: Insights from free N-terminal and mass spectrometry study. Food Chem..

[42] Rolim F.R.L., dos Santos K.M.O., de Barcelos S.C., do Egito A.S., Ribeiro T.S., da Conceição M.L., Magnani M., de Oliveira M.E.G., do Egypto R.D.C.R. (2015). Survival of Lactobacillus rhamnosus EM1107 in simulated gastrointestinal conditions and its inhibitory effect against pathogenic bacteria in semi-hard goat cheese. Lwt.

[43] Sousa R., Portmann R., Dubois S., Recio I., Egger L. (2020). Protein digestion of different protein sources using the INFOGEST static digestion model. Food Res. Int..

[44] Church F.C., Swaisgood H.E., Porter D.H., Catignani G.L. (1983). Spectrophotometric Assay Using o-Phthaldialdehyde for Determination of Proteolysis in Milk and Isolated Milk Proteins. J. Dairy Sci..

[45] Goodno C.C., Swaisgood H.E., Catignani G.L. (1981). A fluorimetric assay for available lysine in proteins. Anal. Biochem..

[46] Bütikofer U., Ardö Y. (1999). Quantitative Determination of Free Amino Acids in cheese. Bull. Int. Dairy Fed..

[47] Duarte V.D.S., Porcellato D. (2024). Host DNA depletion methods and genome-centric metagenomics of bovine hindmilk microbiome. mSphere.

[48] Porcellato D., Meisal R., Bombelli A., Narvhus J.A. (2020). A core microbiota dominates a rich microbial diversity in the bovine udder and may indicate presence of dysbiosis. Sci. Rep..

[49] Bolyen E., Rideout J.R., Dillon M.R., Bokulich N.A., Abnet C.C., Al-Ghalith G.A., Alexander H., Alm E.J., Arumugam M., Asnicar F. (2019). Reproducible, interactive, scalable and extensible microbiome data science using QIIME 2. Nat. Biotechnol..

[50] Callahan B.J., McMurdie P.J., Rosen M.J., Han A.W., Johnson A.J.A., Holmes S.P. (2016). DADA2: High-resolution sample inference from Illumina amplicon data. Nat. Methods..

[51] Hunter J.D. (2007). Matplotlib: A 2D graphics environment. Comput. Sci. Eng..

[52] Seabold S., Perktold J. Econometric and statistical modeling with Python. Proceedings of the 9th Python in Science Conference.

[53] Waskom M.L. (2021). Seaborn: Statistical data visualization. J. Open Source Softw..

[54] Sica J., Bovo B., Nadai C., Carlot M., Giacomini A., Corich V. (2024). Effect of CUP1 copy number and pH on copper resistance of *Saccharomyces cerevisiae* enological strains. Heliyon.

[55] Tjørve K.M.C., Tjørve E. (2017). The use of Gompertz models in growth analyses, and new Gompertz-model approach: An addition to the Unified-Richards family. PLoS ONE.

[56] Virtanen P., Gommers R., Oliphant T.E., Haberland M., Reddy T., Cournapeau D., Burovski E., Peterson P., Weckesser W., Bright J. (2020). SciPy 1.0: Fundamental algorithms for scientific computing in Python. Nat. Methods.

[57] Jakubczyk K., Kałduńska J., Kochman J., Janda K. (2020). Chemical profile and antioxidant activity of the kombucha beverage derived from white, green, black and red tea. Antioxidants.

[58] de Noronha M.C., Cardoso R.R., dos Santos D’Almeida C.T., Vieira do Carmo M.A., Azevedo L., Maltarollo V.G., Júnior J.I.R., Eller M.R., Cameron L.C., Ferreira M.S.L. (2022). Black tea kombucha: Physicochemical, microbiological and comprehensive phenolic profile changes during fermentation, and antimalarial activity. Food Chem..

[59] Jayabalan R., Marimuthu S., Swaminathan K. (2007). Changes in content of organic acids and tea polyphenols during kombucha tea fermentation. Food Chem..

[60] Jayabalan R., Malbaša R.V., Lončar E.S., Vitas J.S., Sathishkumar M. (2014). A review on kombucha tea-microbiology, composition, fermentation, beneficial effects, toxicity, and tea fungus. Compr. Rev. Food Sci. Food Saf..

[61] Ramachandran S., Fontanille P., Pandey A., Larroche C. (2006). Gluconic acid: Properties, applications and microbial production. Food Technol. Biotechnol..

[62] Antolak H., Piechota D., Kucharska A. (2021). Kombucha tea—A double power of bioactive compounds from tea and symbiotic culture of bacteria and yeasts (SCOBY). Antioxidants.

[63] Kallel L., Desseaux V., Hamdi M., Stocker P., Ajandouz E.H. (2012). Insights into the fermentation biochemistry of Kombucha teas and potential impacts of Kombucha drinking on starch digestion. Food Res. Int..

[64] Jafari R., Naghavi N.S., Khosravi-Darani K., Doudi M., Shahanipour K. (2020). Kombucha microbial starter with enhanced production of antioxidant compounds and invertase. Biocatal. Agric. Biotechnol..

[65] Atallah N., Deracinois B., Boulier A., Baniel A., Jouan-Rimbaud Bouveresse D., Ravallec R., Flahaut C., Cudennec B. (2020). *In vitro* assessment of the impact of industrial processes on the gastrointestinal digestion of milk protein matrices using the INFOGEST protocol. Foods.

[66] He Q., Lv Y., Yao K. (2007). Effects of tea polyphenols on the activities of α-amylase, pepsin, trypsin and lipase. Food Chem..

[67] Qie X., Wu Y., Chen Y., Liu C., Zeng M., Qin F., Wang Z., Chen J., He Z. (2021). Competitive interactions among tea catechins, proteins, and digestive enzymes modulate in vitro protein digestibility, catechin bioaccessibility, and antioxidant activity of milk tea beverage model systems. Food Res. Int..

[68] Tantoush Z., Apostolovic D., Kravic B., Prodic I., Mihajlovic L., Stanic-Vucinic D., Cirkovic Velickovic T. (2012). Green tea catechins of food supplements facilitate pepsin digestion of major food allergens, but hampers their digestion if oxidized by phenol oxidase. J. Funct. Foods.

[69] Zhou H., Tan Y., McClements D.J. (2023). Applications of the INFOGEST In Vitro Digestion Model to Foods: A Review. Annu. Rev. Food Sci. Technol..

[70] Alcázar A., Ballesteros O., Jurado J.M., Pablos F., Martín M.J., Vilches J.L., Navalón A. (2007). Differentiation of green, white, black, Oolong, and Pu-erh teas according to their free amino acids content. J. Agric. Food Chem..

[71] Jakubczyk K., Łopusiewicz Ł., Kika J., Janda-milczarek K. (2023). Fermented Tea as a Food with Functional Value—Its Microbiological Profile, Antioxidant Potential and Phytochemical Composition. Foods.

[72] Chen Y., Zeng L., Liao Y., Li J., Zhou B., Yang Z., Tang J. (2020). Enzymatic reaction-related protein degradation and proteinaceous amino acid metabolism during the black tea (*Camellia sinensis*) manufacturing process. Foods.

[73] Jiang X., Ren S., Geng Y., Yu T., Li Y., Liu L., Liu G., Wang H., Shi L. (2020). The sug operon involves in resistance to quaternary ammonium compounds in Listeria monocytogenes EGD-e. Appl. Microbiol. Biotechnol..

[74] Balentine D.A., Wiseman S.A., Bouwens L.C.M. (1997). The Chemistry of Tea Flavonoids. Crit. Rev. Food Sci. Nutr..

[75] Rodriguez Rey J., Tran T., Aumeunier A., Rieu A., Verdier F., Martin A., Alexandre H., Tourdot-Maréchal R., Grandvalet C. (2024). Exploring the role of production and release of proteins for microbial interactions in kombucha. Lwt.

[76] Bishop P., Pitts E.R., Budner D., Thompson-Witrick K.A. (2022). Chemical Composition of Kombucha. Beverages.

[77] Vuong Q.V., Bowyer M.C., Roach P.D. (2011). L-theanine: Properties, synthesis and isolation from tea. J. Sci. Food Agric..

[78] Williams J., Kellett J., Roach P.D., McKune A., Mellor D., Thomas J., Naumovski N. (2016). L-theanine as a functional food additive: Its role in disease prevention and health promotion. Beverages.

[79] Zhao S., Jiao T., Adade S.Y.S.S., Wang Z., Wu X., Ouyang Q., Chen Q. (2024). A rapid method for detecting l-Theanine during kombucha fermentation using SERS combined with machine/deep learning. Microchem. J..

[80] Juneja L.R., Chu D.C., Okubo T., Nagato Y., Yokogoshi H. (1999). L-theanine—A unique amino acid of green tea and its relaxation effect in humans. Trends Food Sci. Technol..

[81] Yamamoto S., Kimura T., Tachiki T., Anzai N., Sakurai T., Ushimaru M. (2012). The involvement of L-type amino acid transporters in theanine transport. Biosci. Biotechnol. Biochem..

[82] Marchese A., Coppo E., Sobolev A.P., Rossi D., Mannina L., Daglia M. (2014). Influence of in vitro simulated gastroduodenal digestion on the antibacterial activity, metabolic profiling and polyphenols content of green tea (*Camellia sinensis*). Food Res. Int..

[83] Zou C., Li R.Y., Chen J.X., Wang F., Gao Y., Fu Y.Q., Xu Y.Q., Yin J.F. (2021). Zijuan tea- based kombucha: Physicochemical, sensorial, and antioxidant profile. Food Chem..

[84] Yamada E.A., Sgarbieri V.C. (2005). Yeast (*Saccharomyces cerevisiae*) protein concentrate: Preparation, chemical composition, and nutritional and functional properties. J. Agric. Food Chem..

[85] Martini A.E.V., Miller M.W., Martini A. (1979). Amino acid composition of whole cells of different yeasts. J. Agric. Food Chem..

[86] Jayabalan R., Malini K., Sathishkumar M., Swaminathan K., Yun S.E. (2010). Biochemical characteristics of tea fungus produced during kombucha fermentation. Food Sci. Biotechnol..

[87] Chu S.C., Chen C. (2006). Effects of origins and fermentation time on the antioxidant activities of kombucha. Food Chem..

[88] Anggraini T., Neswati Nanda R.F., Syukri D. (2021). Effect of Processing on Green and Black Tea DPPH Radical Scavenging Activity, IC50Value, Total Polyphenols, Catechin and Epigallocatechin Gallate content. IOP Conf. Ser. Earth Environ. Sci..

[89] Domínguez-Avila J.A., Wall-Medrano A., Velderrain-Rodríguez G.R., Chen C.Y.O., Salazar-López N.J., Robles-Sánchez M., González-Aguilar G.A. (2017). Gastrointestinal interactions, absorption, splanchnic metabolism and pharmacokinetics of orally ingested phenolic compounds. Food Funct..

[90] Li C.X., Wang F.R., Zhang B., Deng Z.Y., Li H.Y. (2023). Stability and antioxidant activity of phenolic compounds during in vitro digestion. J. Food Sci..

[91] Friedman M., Jürgens H.S. (2000). Effect of pH on the stability of plant phenolic compounds. J. Agric. Food Chem..

[92] Janhavi P., Sindhoora S., Muthukumar S.P. (2020). Bioaccessibility and bioavailability of polyphenols from sour mangosteen (*Garcinia xanthochymus*) fruit. J. Food Meas. Charact..

[93] Mehmood S., Maqsood M., Mahtab N., Khan M.I., Sahar A., Zaib S., Gul S. (2022). Epigallocatechin Gallate: Phytochemistry, Bioavailability, Utilization Challenges, and Strategies. J. Food Biochem..

[94] Ketnawa S., Reginio F.C., Thuengtung S., Ogawa Y. (2022). Changes in bioactive compounds and antioxidant activity of plant-based foods by gastrointestinal digestion: A review. Crit. Rev. Food Sci. Nutr..

[95] Rasera G.B., de Camargo A.C., de Castro R.J.S. (2023). Bioaccessibility of phenolic compounds using the standardized INFOGEST protocol: A narrative review. Compr. Rev. Food Sci. Food Saf..

[96] Kaewkod T., Bovonsombut S., Tragoolpua Y. (2019). Efficacy of kombucha obtained from green, oolongand black teas on inhibition of pathogenic bacteria, antioxidation, and toxicity on colorectal cancer cell line. Microorganisms.

[97] Chakravorty S., Bhattacharya S., Chatzinotas A., Chakraborty W., Bhattacharya D., Gachhui R. (2016). Kombucha tea fermentation: Microbial and biochemical dynamics. Int. J. Food Microbiol..

[98] Tarko T., Duda-Chodak A., Zajac N. (2013). Digestion and absorption of phenolic compounds assessed by in vitro simulation methods. A review. Rocz. Państwowego Zakładu Hig..

[99] Chiang C.J., Kadouh H., Zhou K. (2013). Phenolic compounds and antioxidant properties of gooseberry as affected by in vitro digestion. Lwt.

[100] Liang L., Wu X., Zhao T., Zhao J., Li F., Zou Y., Mao G., Yang L. (2012). In vitro bioaccessibility and antioxidant activity of anthocyanins from mulberry (Morus atropurpurea Roxb.) following simulated gastro-intestinal digestion. Food Res. Int..

[101] Noguer M., Cerezo A.B., Rentzsch M., Winterhalter P., Troncoso A.M., García-Parrilla M.C. (2008). Simulated digestion and antioxidant activity of red wine fractions separated by high speed countercurrent chromatography. J. Agric. Food Chem..

[102] Chen G.L., Chen S.G., Chen F., Xie Y.Q., Han M.D., Luo C.X., Zhao Y.Y., Gao Y.Q. (2016). Nutraceutical potential and antioxidant benefits of selected fruit seeds subjected to an in vitro digestion. J. Funct. Foods.

[103] Stanisavljević N., Samardžić J., Janković T., Šavikin K., Mojsin M., Topalović V., Stevanović M. (2015). Antioxidant and antiproliferative activity of chokeberry juice phenolics during in vitro simulated digestion in the presence of food matrix. Food Chem..

[104] Aspri M., Leni G., Galaverna G., Papademas P. (2018). Bioactive properties of fermented donkey milk, before and after *in vitro* simulated gastrointestinal digestion. Food Chem..

[105] de Campos Costa M.A., de Souza Vilela D.L., Fraiz G.M., Lopes I.L., Coelho A.I.M., Castro L.C.V., Martin J.G.P. (2023). Effect of kombucha intake on the gut microbiota and obesity-related comorbidities: A systematic review. Crit. Rev. Food Sci. Nutr..

[106] Kitwetcharoen H., Phung L.T., Klanrit P., Thanonkeo S., Tippayawat P., Yamada M., Thanonkeo P. (2023). Kombucha Healthy Drink—Recent Advances in Production, Chemical Composition and Health Benefits. Fermentation.

[107] Sanwal N., Gupta A., Bareen M.A., Sharma N., Sahu J.K. (2023). Kombucha fermentation: Recent trends in process dynamics, functional bioactivities, toxicity management, and potential applications. Food Chem. Adv..

[108] Li Y., Wang W., Deng Y., Gao J., Shi J., Cai L. (2024). Antioxidant properties and changes in vitro digestion of the fermented kiwifruit extract prepared by lactic acid bacteria and yeasts. Food Chem..

[109] Nicdao M.A., Ingalla P.C., Ibana J. (2023). *Salmonella enterica* subsp. enterica serovar Typhimurium and *Lactobacillus* spp. interactions in vitro elicit improved antimicrobial production. Trop. Biomed..

[110] Hordofa D.D.L., Nuguse D.A. (2023). Review on Yersiniosis and its public health importance. Int. J. Clin. Biol. Biochem..

[111] Jiang H., Yu F., Qin L., Zhang N., Cao Q., Schwab W., Li D., Song C. (2019). Dynamic change in amino acids, catechins, alkaloids, and gallic acid in six types of tea processed from the same batch of fresh tea (*Camellia sinensis* L.) leaves. J. Food Compos. Anal..

[112] Shad A.A., Shad W.A. (2021). *Shigella sonnei*: Virulence and antibiotic resistance. Arch. Microbiol..

[113] Servin A.L. (2014). Pathogenesis of human diffusely adhering *Escherichia coli* expressing Afa/Dr adhesins (Afa/Dr DAEC): Current insights and future challenges. Clin. Microbiol. Rev..

[114] Smith E.M., Grassel C.L., Papadimas A., Foulke-Abel J., Barry E.M. (2022). The role of cfa/i in adherence and toxin delivery by etec expressing multiple colonization factors in the human enteroid model. PLoS Neglected Trop. Dis..

[115] Bačić A., Gavrilović J., Rajilić-Stojanović M. (2023). Polyphenols as a new class of prebiotics for gut microbiota manipulation. Arh. Farm..

[116] Panzella L., Pérez-Burillo S., Pastoriza S., Martín M.Á., Cerruti P., Goya L., Ramos S., Rufián-Henares J.Á., Napolitano A., D’Ischia M. (2017). High Antioxidant Action and Prebiotic Activity of Hydrolyzed Spent Coffee Grounds (HSCG) in a Simulated Digestion-Fermentation Model: Toward the Development of a Novel Food Supplement. J. Agric. Food Chem..

[117] Bhattacharya D., Bhattacharya S., Patra M.M., Chakravorty S., Sarkar S., Chakraborty W., Koley H., Gachhui R. (2016). Antibacterial Activity of Polyphenolic Fraction of Kombucha Against Enteric Bacterial Pathogens. Curr. Microbiol..

[118] Bhattacharya D., Ghosh D., Bhattacharya S., Sarkar S., Karmakar P., Koley H., Gachhui R. (2018). Antibacterial activity of polyphenolic fraction of kombucha against Vibrio cholerae: Targeting cell membrane. Lett. Appl. Microbiol..

[119] Sreeramulu G., Zhu Y., Knol W. (2000). Kombucha fermentation and its antimicrobial activity. J. Agric. Food Chem..

[120] Al-Mohammadi A.R., Ismaiel A.A., Ibrahim R.A., Moustafa A.H., Abou Zeid A., Enan G. (2021). Chemical constitution and antimicrobial activity of kombucha fermented beverage. Molecules.

[121] Sreeramulu G., Zhu Y., Knol W. (2001). Characterization of antimicrobial activity in Kombucha fermentation. Acta Biotechnol..

[122] Thenuwara G., Cui X., Yao Z., Javed B., Naik A.S., Tian F. (2024). Evaluating the Health Implications of Kombucha Fermented with *Gardenia jasminoides* Teas: A Comprehensive Analysis of Antioxidant, Antimicrobial, and Cytotoxic Properties. BioChem.

[123] Toda M., Okubo S., Ikigai H., Suzuki T., Suzuki Y., Shimamura T. (1991). The protective activity of tea against infection by Vibrio cholerae O1. J. Appl. Microbiol..

[124] Pei J., Jin W., Abd El-Aty A.M., Baranenko D.A., Gou X., Zhang H., Geng J., Jiang L., Chen D., Yue T. (2020). Isolation, purification, and structural identification of a new bacteriocin made by Lactobacillus plantarum found in conventional kombucha. Food Control..

[125] Breijyeh Z., Jubeh B., Karaman R. (2020). Resistance of Gram-Negative Bacteria to Current Antibacterial Agents and Approaches to Resolve It. Molecules.

[126] Battikh H., Bakhrouf A., Ammar E. (2012). Antimicrobial effect of Kombucha analogues. Lwt.

[127] Wang B., Rutherfurd-Markwick K., Liu N., Zhang X.X., Mutukumira A.N. (2024). Evaluation of the probiotic potential of yeast isolated from kombucha in New Zealand. Cur. Res. Food Sci..

[128] Minekus M., Alminger M., Alvito P., Ballance S., Bohn T., Bourlieu C., Carrière F., Boutrou R., Corredig M., Dupont D. (2014). A standardised static in vitro digestion method suitable for food-an international consensus. Food Funct..

[129] de Oliveira M.E.G., Garcia E.F., de Oliveira C.E.V., Gomes A.M.P., Pintado M.M.E., Madureira A.R.M.F., da Conceição M.L., do EgyptoQueiroga R.D.C.R., de Souza E.L. (2014). Addition of probiotic bacteria in a semi-hard goat cheese (coalho): Survival to simulated gastrointestinal conditions and inhibitory effect against pathogenic bacteria. Food Res. Int..

[130] Sharifudin S.A., Ho W.Y., Yeap S.K., Abdullah R., Koh S.P. (2021). Fermentation and characterisation of potential kombucha cultures on papaya-based substrates. Lwt.

[131] Harrison K., Curtin C. (2021). Microbial composition of scoby starter cultures used by commercial kombucha brewers in North America. Microorganisms.

[132] Laavanya D., Shirkole S., Balasubramanian P. (2021). Current challenges, applications and future perspectives of SCOBY cellulose of kombucha fermentation. J. Clean. Prod..

[133] Aung T., Kim M.J. (2024). A comprehensive review on kombucha biofilms: A promising candidate for sustainable food product development. Trends Food Sci. Technol..

[134] Yamada Y., Yukphan P., Vu H.T.L., Muramatsu Y., Ochaikul D., Tanasupawat S., Nakagawa Y. (2012). Description of *Komagataeibacter* gen. nov., with proposals of new combinations (Acetobacteraceae). J. Gen. Appl. Microbiol..

[135] Coton M., Pawtowski A., Taminiau B., Burgaud G., Deniel F., Coulloumme-Labarthe L., Fall A., Daube G., Coton E. (2017). Unraveling microbial ecology of industrial-scale kombucha fermentations by metabarcoding and culture-based methods. FEMS Microbiol. Ecol..

[136] Neffe-Skocińska K., Długosz E., Szulc-Dąbrowska L., Zielińska D. (2024). Novel *Gluconobacter* oxydans strains selected from kombucha with potential postbiotic activity. Appl. Microbiol. Biotechnol..

[137] Food and Agriculture Organization of the United Nations, World Health Organization (2006). Probiotics in food: Health and nutritional properties and guidelines for evaluation. FAO Food and Nutritional Paper No. 85.

[138] Hill C., Guarner F., Reid G., Gibson G.R., Merenstein D.J., Pot B., Morelli L., Canani R.B., Flint H.J., Salminen S. (2014). The international scientific association for probiotics and prebiotics consensus statement on the scope and appropriate use of the term probiotic. Nat. Rev. Gastroenterol. Hepatol..

[139] Sanders M.E., Merenstein D.J., Reid G., Gibson G.R., Rastall R.A. (2019). Probiotics and prebiotics in intestinal health and disease: From biology to the clinic. Nat. Rev. Gastroenterol. Hepatol..

